# De novo transcriptome assembly of *Conium maculatum* L. to identify candidate genes for coniine biosynthesis

**DOI:** 10.1038/s41598-022-21728-w

**Published:** 2022-10-20

**Authors:** Gopal Peddinti, Hannu Hotti, Teemu H. Teeri, Heiko Rischer

**Affiliations:** 1 VTT Technical Research Centre of Finland Ltd, Tietotie 2, VTT, P.O. Box 1000, 02044 Espoo, Finland; 2 Viikki Plant Science Centre, Department of Agricultural Sciences, University of Helsinki, PO Box 27, 00014 Helsinki, Finland; 3 Present Address: Faculty of Biological and Environmental Sciences, University of Helsinki, PO Box 56, 00014 Helsinki, Finland

**Keywords:** Transcriptomics, Secondary metabolism

## Abstract

Poison hemlock (*Conium maculatum* L.) is a notorious weed containing the potent alkaloid coniine. Only some of the enzymes in the coniine biosynthesis have so far been characterized. Here, we utilize the next-generation RNA sequencing approach to report the first-ever transcriptome sequencing of five organs of poison hemlock: developing fruit, flower, root, leaf, and stem. Using a de novo assembly approach, we derived a transcriptome assembly containing 123,240 transcripts. The assembly is deemed high quality, representing over 88% of the near-universal ortholog genes of the Eudicots clade. Nearly 80% of the transcripts were functionally annotated using a combination of three approaches. The current study focuses on describing the coniine pathway by identifying in silico transcript candidates for polyketide reductase, l-alanine:5-keto-octanal aminotransferase, γ-coniceine reductase, and *S*-adenosyl-l-methionine:coniine methyltransferase. In vitro testing will be needed to confirm the assigned functions of the selected candidates.

## Introduction

Poison hemlock (*Conium maculatum* L.) is a well-known poisonous plant^1^ native to Europe which has spread as an invasive plant to the Americas and Australia^2^. It is an old medicinal plant not currently used due to a narrow treatment window^2^. Poison hemlock is notorious for containing potent alkaloids, i.e. coniine and its derivatives^1^. Currently, it is uncertain whether such alkaloids occur in other Apiaceae, e.g.* Pimpinella acuminata*^3^. Coniine-type alkaloids have otherwise only been confirmed in unrelated taxa, i.e. seven species of *Sarracenia*^4^ and twelve *Aloe*^5–8^.

**Figure 1:**
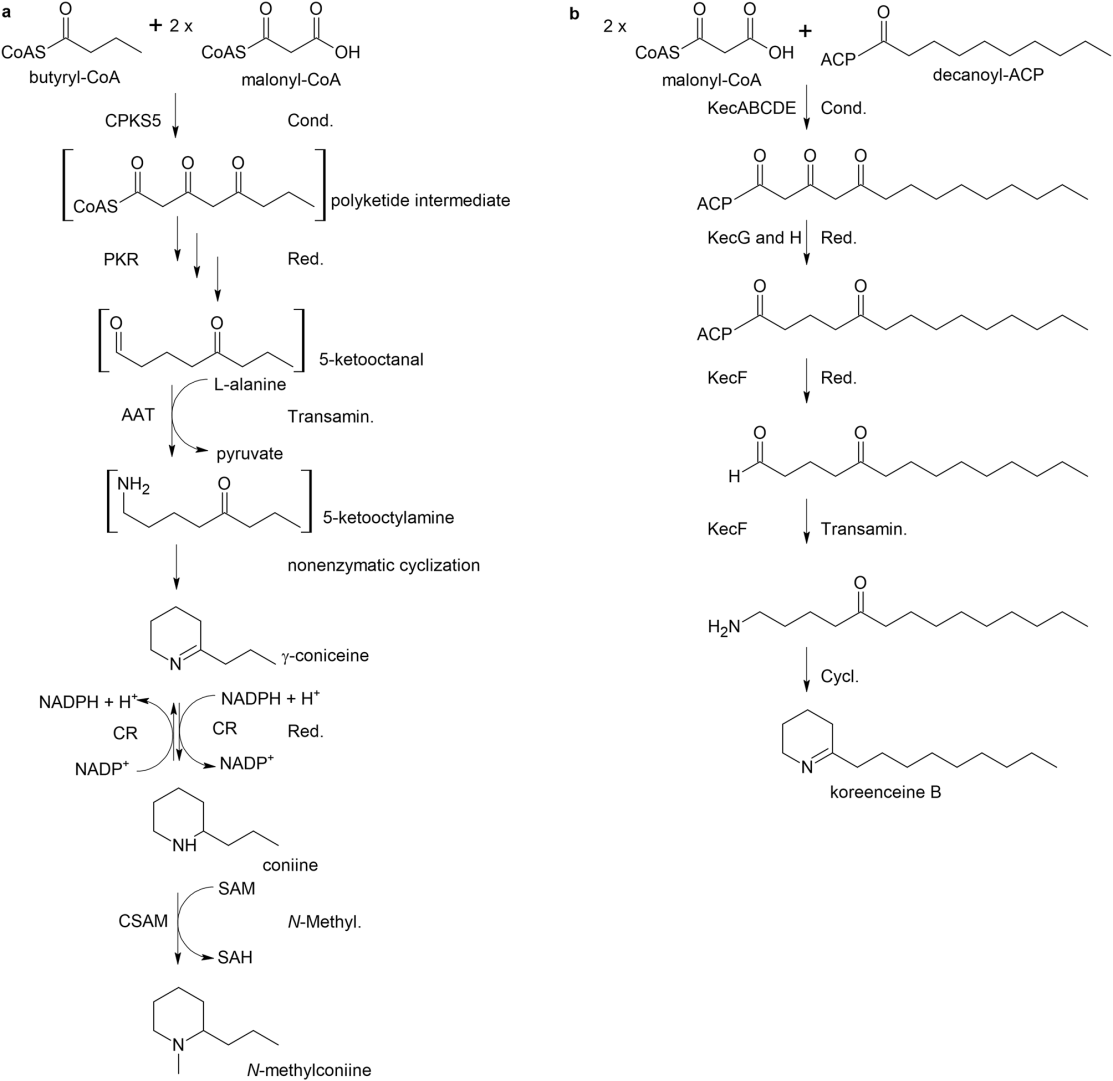
(**a**) Proposed biosynthesis pathway of coniine in poison hemlock (*Conium maculatum* L.) according to Hotti and Rischer^1^. Abbreviations: AAT l-alaninen:5-ketooctanal aminotransferase, CR γ-coniceine reductase, CSAM *S*-adenosyl-l-methionine:coniine methyltransferase, SAH *S*-adenosyl-l-homocysteine, SAM *S*-adenosyl-l-methionine. (**b**) Biosynthesis pathway of koreenceine in *Pseudomonas koreensis*^20^*.* The ChemSketch Freeware version 2021 (https://www.acdlabs.com/resources/free-chemistry-software-apps/chemsketch-freeware/) was used to draw this figure.

Previous research efforts have led to a proposed biosynthetic pathway supported by experimental evidence concerning individual reactions and active enzymes. Accordingly, the pathway commences with the formation of the carbon backbone. A type III polyketide synthase (PKS), *Conium* polyketide synthase 5 (CPKS5)^9^, forms a triketide product from one butyryl-CoA and two malonyl-CoAs (Fig. 1A). The next hypothetical step is a reduction of the polyketide by a polyketide reductase (PKR) to form 5-keto-octanal. Subsequently, a transamination reaction by l-alanine:5-keto-octanal aminotransferase (AAT) transfers nitrogen from l-alanine^10^. Next, γ-coniceine is formed by a non-enzymatic reaction^11,12^. Coniine is then formed by an NADPH-dependent γ-coniceine reductase (CR)^13^. The final step is the formation of *N*-methylconiine by *S*-adenosyl-l-methionine:coniine methyltransferase (CSAM), for which *S*-adenosyl-l-methionine (SAM) is the donor of a methyl group^14,15^.

The content of coniine and other related alkaloids in different tissues has been extensively studied and reported in the literature (e.g. Fairbairn and Suwal^16^). The concentrations of coniine and γ-coniceine fluctuate depending on the time of the day and weather^16,17^. Coniine alkaloids are found in above-ground parts, however, Cromwell^18^ and Fairbairn and Suwal^16^ noted that the roots of poison hemlock contain alkaloids only after 1st year of growth. Roberts^19^ noticed that key enzymes-AAT, CR, and CSAM-are active during leaf expansion and their activity ceases when the leaf is fully grown.

In a study of the microbial community in the soybean (*Glycine max* (L.) Merr) rhizosphere, Lozano and colleagues^20^ identified *Pseudomonas koreensis*^21^ genes involved in the biosynthesis of a new family of four bacterial alkaloids, named koreenceine A to D (Fig. 1B), three of which are analogues of the plant alkaloid γ-coniceine. The bacterial koreenceine biosynthetic pathway bears considerable similarities to the coniine pathway in plants, although it involves a type II PKS instead of a type III PKS. However, the other bacterial enzymes in the pathway may help identify γ-coniceine biosynthesis enzymes in the *C. maculatum* transcriptome.

The next-generation sequencing approach known as massively parallel complementary DNA sequencing (RNA-seq) is typically used for analysing the transcriptome of an organism in two ways: (1) when an annotated reference genome assembly for the organism is available, the sequencing reads are aligned to the reference assembly to quantify the gene expression values, and (2) for non-model organisms without a reference genome assembly, the transcriptomes are reconstructed using de novo transcriptome assembly algorithms, making it possible to explore all expressed genes^22,23^.

In this study, we present a de novo transcriptome assembly based on RNA-sequencing of five different organs of *C. maculatum*. Currently, the only known sequence for an enzyme involved in the coniine biosynthesis is CPKS5^9^, catalysing the first committed step. Other enzymes are only enzymatically characterised, the protein size^11^ has been determined, and in the case of ATT, the isoform expression and targeting locations (chloroplast and mitochondria of the leaf)^24^ are known. Therefore, we hypothesize that a de novo assembly of the poison hemlock transcriptome would allow us to perform an in silico selection of candidates for the remaining enzymes in the biosynthetic pathway, namely, for AAT, CR, and CSAM. The de novo assembly and annotation presented in this study additionally allows the analysis of the global gene expressions of *C. maculatum*.

## Results

### Transcriptome assembly and overall expression patterns

**Figure 2:**
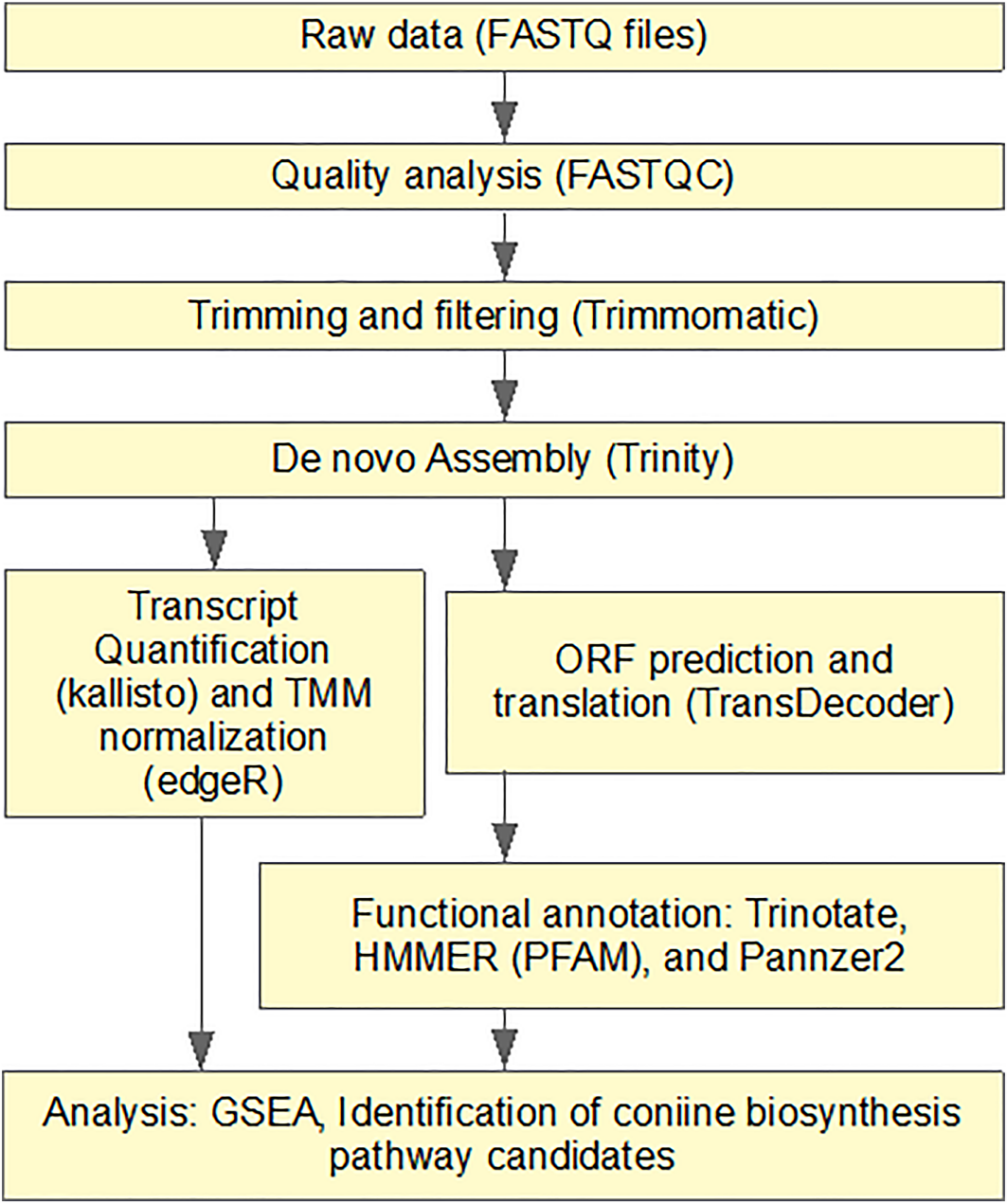
Outline of the data processing. The LibreOffice Draw v6.4 (https://www.libreoffice.org/discover/draw/) was used to draw this figure.

**Figure 3:**
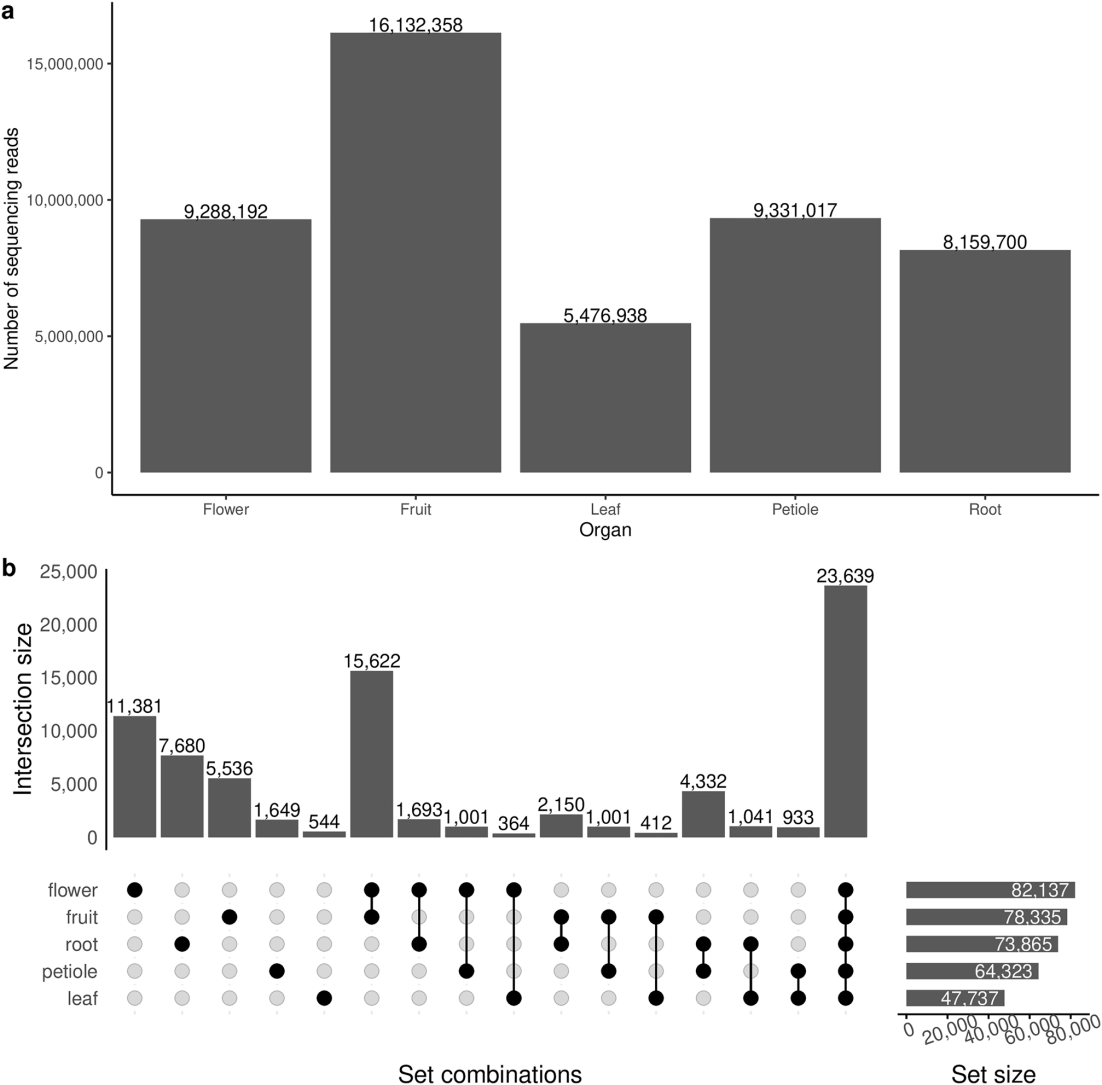
Data distribution across various organs of *Conium maculatum* L. (**a**) Illumina read set sizes: the bar shows the number of paired-end sequencing reads in each organ by averaging the number of reads among the two replicates. (**b**) Assembled transcripts: the bars indicate the numbers of transcripts expressed in each organ by averaging the expression value between replicates. The R packages *ggplot* (https://ggplot2.tidyverse.org) and *ComplexUpset* (http://doi.org/10.5281/zenodo.3700590) were used to draw these figures.

The raw NGS reads of the transcriptomic samples contained 0.5–17.4 million pairs of 140 bp reads, processing of which is summarized in Fig. 2. The distribution of the average number of reads per organ is shown in Fig. 3a. The pooled transcriptome assembly of all organs consisted of 179,808 transcripts. Of these, 56,568 transcripts (31%) were identified as originating from bacteria, fungi or animals such as insects and designated as contaminant transcripts. The contaminants were distributed among the organs as follows. The largest contaminants (n = 10,742) were exclusively expressed in the root. This was followed by flower (n = 8183) and fruit (n = 3892). All the organs expressed a common set of 5669 contaminant transcripts. The decontaminated assembly that excluded the contaminant transcripts consisted of 123,240 transcripts (Supplementary Data S1). Lengths of the transcripts in the decontaminated transcriptome ranged from 297 to 14,883 nucleotides with a median length of 1500 nucleotides. Out of the expected number of 2326 universal single-copy orthologs for the taxonomic clade of Eudicots, 2050 complete genes (88.1%) were found in the transcriptome assembly (BUSCO quality: C: 88.1% [S: 15.1%, D: 73.0%], F: 3.5%, M: 8.4%, n: 2 326).

Out of the 123,240 transcripts in the transcriptome assembly, a total of 98,003 transcripts (79.5%) were annotated. Of these, Pannzer2^25^ predicted gene definitions for 46,263 (37.5%) transcripts, Trinotate^26^ annotated 14,681 (11.9%) transcripts with Uniprot^27^ protein definitions, and HMMER^28^ annotated 61,733 (50%) transcripts with PFAM protein family definitions. Pannzer2 and Trinotate annotated 74,471 (60%) transcripts with gene ontology biological process terms and 17,772 (14%) transcripts with KEGG metabolic pathways^29^. The enzyme annotations included 929 fully determined enzyme commission (EC) numbers. These EC numbers covered 814 of the 2756 enzymes represented in the KEGG global metabolic network (KEGG pathway *map01100 metabolic pathways*, Supplementary Fig. S1) and 434 of the 1321 enzymes represented in the KEGG network of secondary metabolite biosynthesis (*map01110 biosynthesis of secondary metabolites*, Supplementary Fig. S2). The distribution of the enzymes across the metabolic pathways (sub-networks) is presented in Supplementary Table S2.

The number of transcripts expressed in each organ of the *C. maculatum* is shown in Fig. 3b. The flower expressed the largest number of transcripts (n = 82,137), followed by fruit (n = 78,335), root (n = 73,865), petiole (n = 64,323), and leaf (n = 47,737) in that order. A shared set of 23,639 transcripts were expressed in all organs. The flower expressed the largest number of unique transcripts not expressed in other organs (n = 11,381), followed by root (n = 7680), fruit (n = 5536), petiole (n = 1649), and leaf (n = 544). A shared set of 61,870 transcripts were expressed in flower and fruit, and among these shared transcripts, 15,622 were exclusively expressed in only fruit and flower. Root and petiole shared the next largest set of exclusively expressed common transcripts (n = 4332). Similarly, the flower and leaf shared the smallest set of transcripts exclusively expressed in them (n = 364).

**Figure 4:**
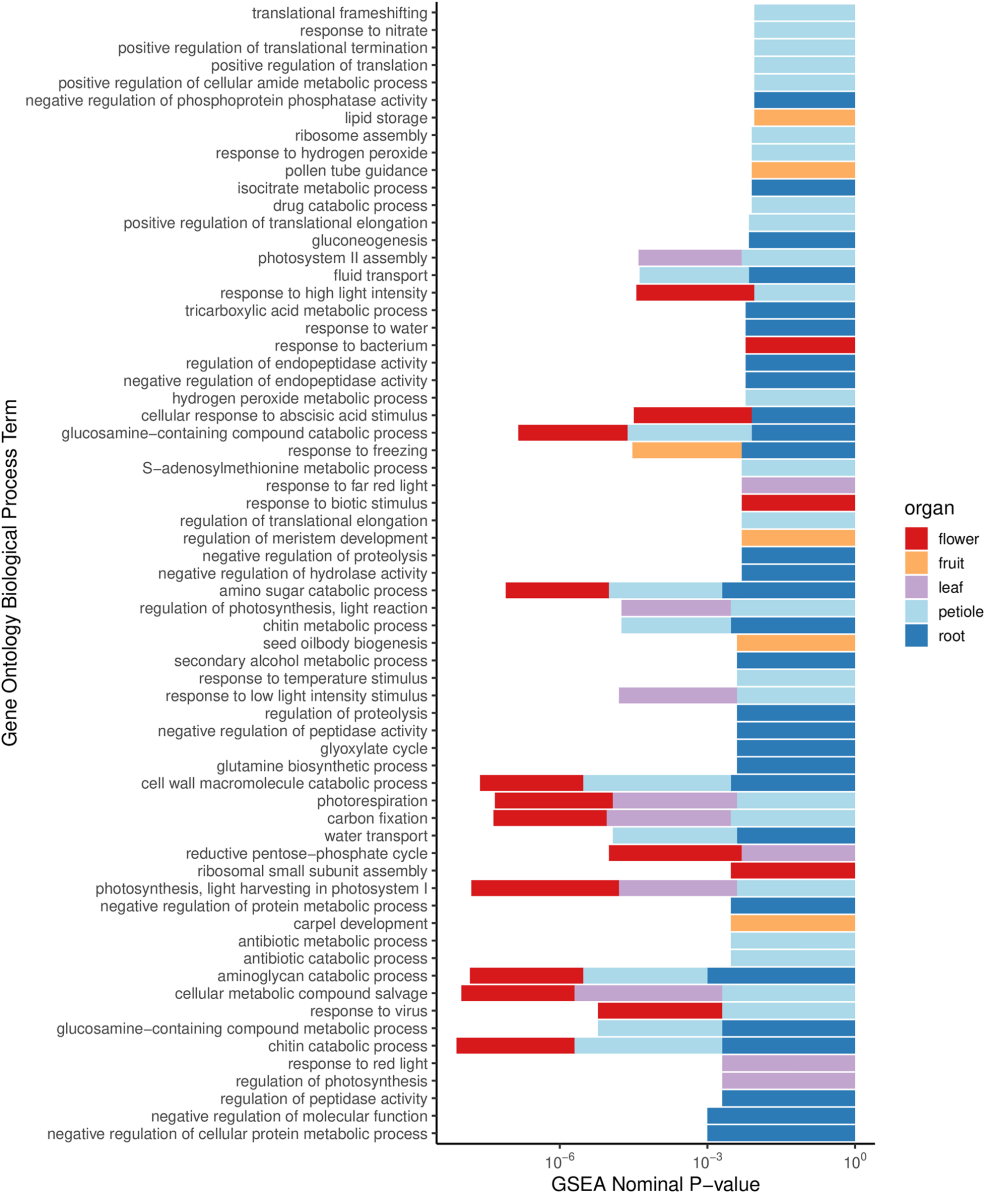
Gene ontology biological process terms in the genes expressed in each organ, found using gene set enrichment analysis (nominal *p* value < 0.01). The height of the bar reflects the negative logarithm (base 10) of the p value, and the colours are used to indicate the organs. The R package *ggplot* (https://ggplot2.tidyverse.org) was used to draw this figure.

To understand the biological functions of the genes expressed in each organ, we performed gene set enrichment analysis (GSEA)^30^ with GO biological process terms as the gene categories. Using a nominal *p* value cut-off of 0.01, we found 50 GO terms represented in *C. maculatum* flower, 8 in developing fruit, 15 in leaf, 62 in the root, and 51 in the petiole. The GO terms that contained less than 15 were removed from the analysis. The complete list of GO biological process terms and their GSEA statistics are provided in Supplementary Data S2. The GO terms containing less than 100 transcripts are shown in Fig. 4. Finally, the expression patterns of the genes involved in the plant hormone biosynthesis (KEGG ID: *map01070*) have been provided via the five sub-pathways of the plant hormone biosynthesis, namely, fatty acid biosynthesis (Supplementary Fig. S3), terpenoid backbone biosynthesis (Supplementary Fig. S4), brassinosteroid biosynthesis (Supplementary Fig. S5), zeatin biosynthesis (Supplementary Fig. S6) and biosynthesis of unsaturated fatty acids (Supplementary Fig. S7).

### Identification of the coniine biosynthesis pathway enzymes

#### Type III polyketide synthases (CPKS5)

**Figure 5:**
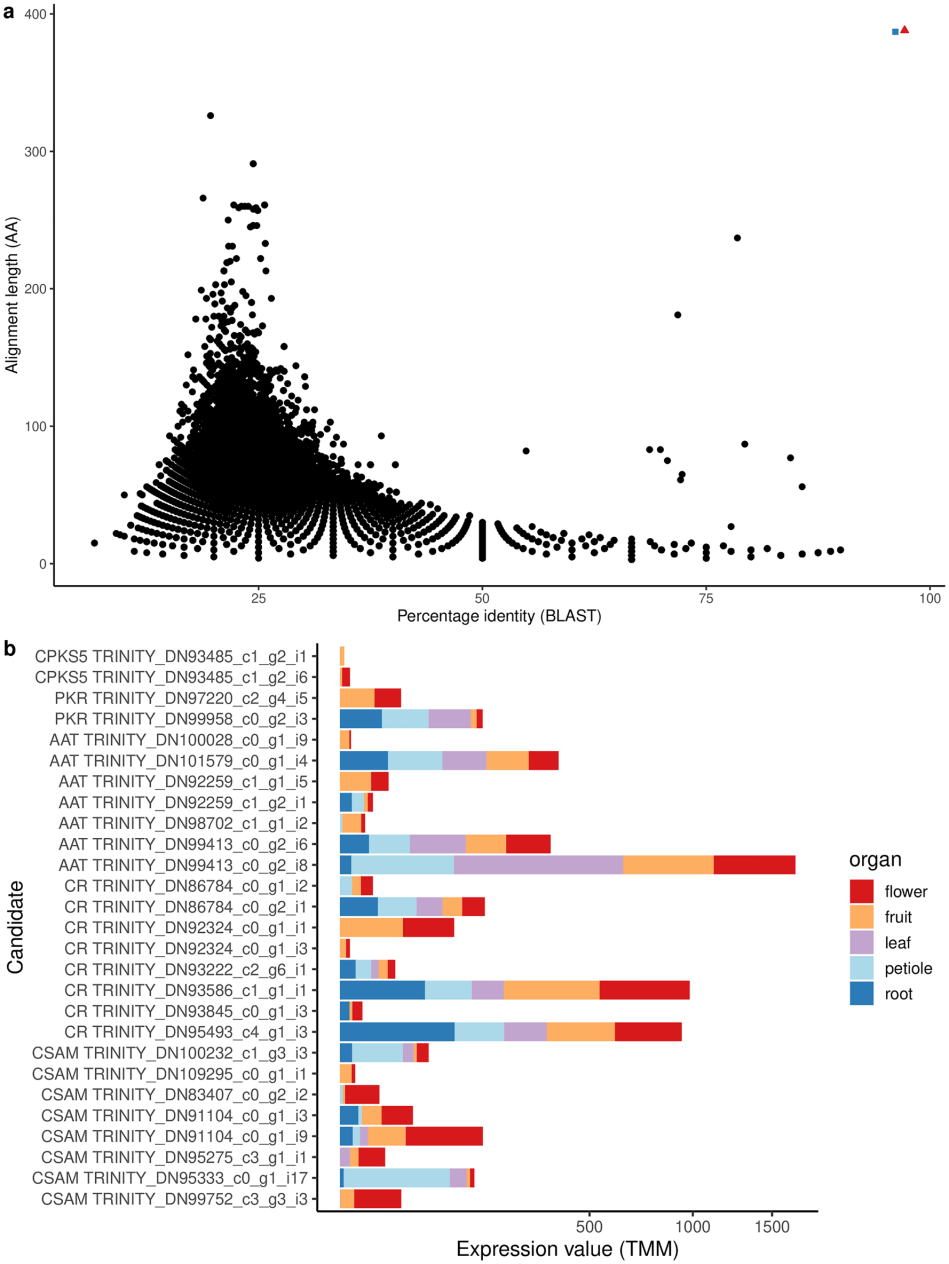
The transcript candidates potentially encode *Conium* polyketide synthase 5 (CPKS5) and the other enzymes of the coniine pathway. (**a**) BLAST search results presented as a scatterplot between percentage identity and alignment length in amino acids. The best matching transcript candidates in terms of sequence similarity and alignment length are highlighted in the graph (red triangle, TRINITY_DN93485_c1_g2_i1; blue square, TRINITY_DN93485_c1_g2_i6). (**b**) The expression values (TMM) of selected enzyme candidates. The R package *ggplot* (https://ggplot2.tidyverse.org) was used to draw this figure.

The peptide sequences predicted from the transcriptome assembly were queried with protein-BLAST using the known CPKS5 sequence. The BLAST hits were visualised as a scatter plot with percentage identity as the x-axis and the alignment length as the y-axis (Fig. 5) to aid in picking the candidates with the highest sequence homology. The best matching hits would show high sequence identity with high sequence length coverage in the alignment and hence would appear along the top-right corner with the highest combination of x- and y-coordinate values. Two full-length transcripts, TRINITY_DN93485_c1_g2_i1 and TRINITY_DN93485_c1_g2_i6, were found with these sequence homology criteria to be the CPKS5 candidates in the *C. maculatum* assembly. These two candidates differed from each other by four amino acids in C-terminus. Compared to the previously published CPKS5 sequence^9^, the current candidates differed by eleven and fifteen amino acids, respectively, but had identical amino acids in the catalytic centre (Supplementary Data S3).

#### l-Alanine:5-ketooctanal aminotransferase (AAT)

Based on sequence homology searches (i.e., BLAST searches with known aminotransferase sequences and HMM search for aminotransferase protein families), 302 aminotransferase candidates were found, of which 137 transcripts were expressed in flower and developing fruit similarly to CPKS5, and 48 of them were predicted to have a molecular weight in the range of 45 kDa to 65 kDa (i.e., within a 10 kDa window around the 56.2 kDa determined by Roberts^11^). From the 48 candidates within the correct weight range, the functional annotations were used to filter the candidate list by removing the transcripts whose functions are irrelevant to the alanine transamination, leading to the selection of six candidates comprised of seven transcripts annotated to be participating in alanine transamination (Table 1).

**Table 1:** Top in silico candidates for PKR, AAT, CR, and CSAM of coniine biosynthesis.

Enzyme	Transcript ID	E.C	PFAM	Size (bp)	Size (aa)	Size (kDa)	Nearest NCBI-BLAST hit
PKR	PKR1: TRINITY_DN97220_c2_g4_i5	1.1.1.100; 2.3.1.85	PF00106.23 short chain dehydrogenase; PF13561.4 Enoyl-(Acyl carrier protein) reductase; PF08659.8 KR domain	1194	398	42.29	*Daucus carota* subsp. *sativus* 3-oxoacyl-[acyl-carrier-protein] reductase 4-like (LOC108197291), mRNA
	PKR2: TRINITY_DN99958_c0_g2_i3	1.1.1.100; 2.3.1.85	PF00106.23 short chain dehydrogenase; PF13561.4 Enoyl-(Acyl carrier protein) reductase; PF08659.8 KR domain	963	321	33.39	*D. carota* subsp. *sativus* 3-oxoacyl-[acyl-carrier-protein] reductase 4-like (LOC108197291), mRNA
AAT	AAT1: TRINITY_DN100028_c0_g1_i9	2.6.1.4; 2.6.1.2; 2.6.1.44; 2.6.1.12	PF00155.19 Aminotransferase class I and II	1443	481	53.27	*D. carota* subsp. *sativus* glutamate–glyoxylate aminotransferase 2 (LOC108194271), transcript variant X1, mRNA
	AAT2: TRINITY_DN101579_c0_g1_i4	2.6.1.2; 2.6.1.12	PF00155.19 Aminotransferase class I and II	1638	546	60.01	*D. carota* subsp. *sativus* alanine aminotransferase 2-like (LOC108222962), mRNA
	AAT3: TRINITY_DN92259_c1_g1_i5/ TRINITY_DN92259_c1_g2_i1		PF00202.19 Aminotransferase class-III	1422	474	51.76	*D. carota* subsp. *sativus* alanine–glyoxylate aminotransferase 2 homolog 1, mitochondrial (LOC108217775), transcript variant X1, mRNA
	AAT4: TRINITY_DN98702_c1_g1_i2	2.6.1.96; 2.6.1.62	PF00202.19 Aminotransferase class-III	1413	471	52.13	*D. carota* subsp. *sativus* gamma aminobutyrate transaminase 2-like (LOC108200542), mRNA
	AAT5: TRINITY_DN99413_c0_g2_i6	2.6.1.4; 2.6.1.2; 2.6.1.44; 2.6.1.12	PF00155.19 Aminotransferase class I and II	1437	479	52.81	*D. carota* subsp. *sativus* glutamate–glyoxylate aminotransferase 2 (LOC108210226), mRNA
	AAT6: TRINITY_DN99413_c0_g2_i8	2.6.1.4; 2.6.1.2; 2.6.1.44; 2.6.1.12	PF00155.19 Aminotransferase class I and II	1437	479	52.95	*D. carota* subsp. *sativus* glutamate–glyoxylate aminotransferase 2-like (LOC108202675), transcript variant X1, mRNA
CR	CR1: TRINITY_DN86784_c0_g1_i2/ TRINITY_DN86784_c0_g2_i1		PF13561.4 Enoyl-(Acyl carrier protein) reductase; PF00106.23 short chain dehydrogenase	912	304	32.06	*D. carota* subsp. *sativus* peroxisomal 2,4-dienoyl-CoA reductase-like (LOC108206087), mRNA
	CR2: TRINITY_DN92324_c0_g1_i1		PF13561.4 Enoyl-(Acyl carrier protein) reductase; PF00106.23 short chain dehydrogenase	891	297	31.44	*D. carota* subsp. *sativus* peroxisomal 2,4-dienoyl-CoA reductase (LOC108224824), mRNA
	CR3: TRINITY_DN92324_c0_g1_i3		PF13561.4 Enoyl-(Acyl carrier protein) reductase; PF00106.23 short chain dehydrogenase	741	247	26.03	*D. carota* subsp. *sativus* peroxisomal 2,4-dienoyl-CoA reductase (LOC108224824), mRNA
	CR4: TRINITY_DN93222_c2_g6_i1	1.3.1.33; 1.1.1.2	PF00106.23 short chain dehydrogenase	732	244	27.19	*D. carota* subsp. *sativus* short-chain dehydrogenase TIC 32, chloroplastic-like (LOC108211237)
	CR5: TRINITY_DN93586_c1_g1_i1	1.1.1.330; 1.3.1.33	PF00106.23 short chain dehydrogenase	945	315	34.29	*D. carota* subsp. *sativus* short-chain dehydrogenase TIC 32, chloroplastic-like (LOC108196149), mRNA
	CR6: TRINITY_DN93845_c0_g1_i3	1.3.1.33; 1.1.1.330	PF00106.23 short chain dehydrogenase	636	212	22.86	*D. carota* subsp. *sativus* short-chain dehydrogenase TIC 32, chloroplastic-like (LOC108199832), mRNA
	CR7: TRINITY_DN95493_c4_g1_i3	1.1.1.330; 1.3.1.33	PF00106.23 short chain dehydrogenase	771	257	28.25	*D. carota* subsp. *sativus* short-chain dehydrogenase TIC 32, chloroplastic-like (LOC108211237)
CSAM	CSAM1: TRINITY_DN100232_c1_g3_i3		PF03492.13 SAM dependent carboxyl methyltransferase	399	133	15.21	*D. carota* subsp. *sativus* benzoate carboxyl methyltransferase-like (LOC108212050), mRNA
	CSAM2: TRINITY_DN109295_c0_g1_i1			492	164	18.78	No hits
	CSAM3: TRINITY_DN83407_c0_g2_i2		PF03492.13 SAM dependent carboxyl methyltransferase	474	158	17.82	*D. carota* subsp. *sativus* benzoate carboxyl methyltransferase-like (LOC108202053), mRNA
	CSAM4: TRINITY_DN91104_c0_g1_i3		PF03492.13 SAM dependent carboxyl methyltransferase	1107	369	41.43	*D. carota* subsp. *sativus* salicylate carboxymethyltransferase-like (LOC108225466), mRNA
	CSAM5: TRINITY_DN91104_c0_g1_i9		PF03492.13 SAM dependent carboxyl methyltransferase	669^a^	223^a^	25.37^a^	*D. carota* subsp. *sativus* salicylate carboxymethyltransferase-like (LOC108225576), mRNA
	CSAM6: TRINITY_DN95275_c3_g1_i1/ TRINITY_DN99752_c3_g3_i3		PF03492.13 SAM dependent carboxyl methyltransferase	1071	357	40.43	*D. carota* subsp. *sativus* salicylate carboxymethyltransferase-like (LOC108194344), mRNA
	CSAM7: TRINITY_DN95333_c0_g1_i17		PF03492.13 SAM dependent carboxyl methyltransferase	438	146	16.39	*D. carota* subsp. *sativus* benzoate carboxyl methyltransferase-like (LOC108212050), mRNA

Transcript ID shows the identifier of the transcript obtained in Trinity assembly. Enzyme commission number (E.C.) and protein family identifier (PFAM) were derived using the annotation approach described in the manuscript. The Size (bp) and Size (aa) show the size of the transcript in terms of nucleotide and predicted amino acid sequence. The molecular weight was predicted from the peptide sequence as described in the manuscript.

^a^Incomplete sequence.

Three among the seven AAT transcripts selected (TRINITY_DN92259_c1_g2_i1, TRINITY_DN92259_c1_g1_i5 and TRINITY_DN98702_c1_g1_i2) belong to the Aminotransferase Class-III protein family (PFAM PF00202), as the koreenceine transaminase *kecF* of *P. koreensis*^20^. The transcript TRINITY_DN98702_c1_g1_i2 was found in the HMM search for the protein family as well as BLAST, while TRINITY_DN92259_c1_g2_i1 and TRINITY_DN92259_c1_g1_i5 were found solely in the protein family search.

#### Reductases (PKR and CR)

As a PKR is needed in-between reactions catalysed by CPKS5 and AAT, we looked for a PKR based on the *P. koreensis* ketoreductase kecG^20^, which has a KR domain (PFAM id: PF08659). With protein family search using hidden Markov models, we found two in silico candidates, TRINITY_DN97220_c2_g4_i5 and TRINITY_DN99958_c0_g2_i3, containing the KR domain and expressed in flower and developing fruit like CPKS5 (Table 1).

Using the protein families found in the NAD(P)H oxidoreductase kecH^20^ (Table 2) and expression in flower and developing fruit as the criteria, we found 128 CR candidates. These were further analysed and categorised into three groups based on whether they used NADPH, NAPDH/NADH, or NADH as cofactors. As Roberts^13^ enzymatically characterised CR and noted that the enzyme uses NADPH as the cofactor, we excluded 23 candidates utilising NADH. Next, we checked 30 candidates which were annotated to use only NADPH. Among them, two candidates belonged to CH-CH oxidoreductase class of enzymes (E.C. number 1.3.1), TRINITY_DN93586_c1_g1_i1 and TRINITY_DN95493_c4_g1_i3, which are our top candidates for CR. The NADPH/NADH groups contain 77 transcripts, of which six have E.C. 1.3.1 numbering (Table 1).

**Table 2:** The number of transcripts sharing protein family domains with the bacterial koreenceine biosynthesis pathway.

The bacterial gene involved in Koreenceine synthesis	Function predicted by Lozano et al.^20^	PFAM id	PFAM description	Number of *Conium maculatum* transcripts sharing PFAM domain
kecF	Pyridoxalphosphate-dependent aminotransferase	PF00202	Aminotransferase class-III	81
kecG	Ketoreductase	PF00106	Short-chain dehydrogenase	436
kecG	Ketoreductase	PF08659	KR domain	14
kecG	Ketoreductase	PF13561	Enoyl-(Acyl carrier protein) reductase	331
kecH	NAD(P)H oxidoreductase	PF00106	Short-chain dehydrogenase	436
kecH	NAD(P)H oxidoreductase	PF08659	KR domain	14
kecH	NAD(P)H oxidoreductase	PF13561	Enoyl-(Acyl carrier protein) reductase	331

As the plant pathway uses PKS type III, unlike the bacterial pathway, we have excluded the PKS genes and included only the polyketide accessory genes and transaminase in our analyses when comparing the bacterial genes with *C. maculatum* transcripts.

#### SAM-dependent methyltransferase (CSAM)

As the CSAM is the final enzyme on the biosynthesis route transforming coniine to *N*-methylconiine, SAM is required^14,15^. We found nine SAM-dependent methyltransferase transcripts that were expressed in flower and developing fruit (Table 1). Eight of these nine transcripts belong to the PFAM group PF03492 (SAM-dependent carboxyl methyltransferases).

Finally, it should be noted that based on sequence similarity, no evidence for the involvement of a polyketide cyclase to form gamma-coniceine after transamination analogously to olivetolic acid cyclase in the cannabinoid pathway^31^ could be found.

#### Confirmation of expression patterns by quantitative PCR

**Figure 6:**
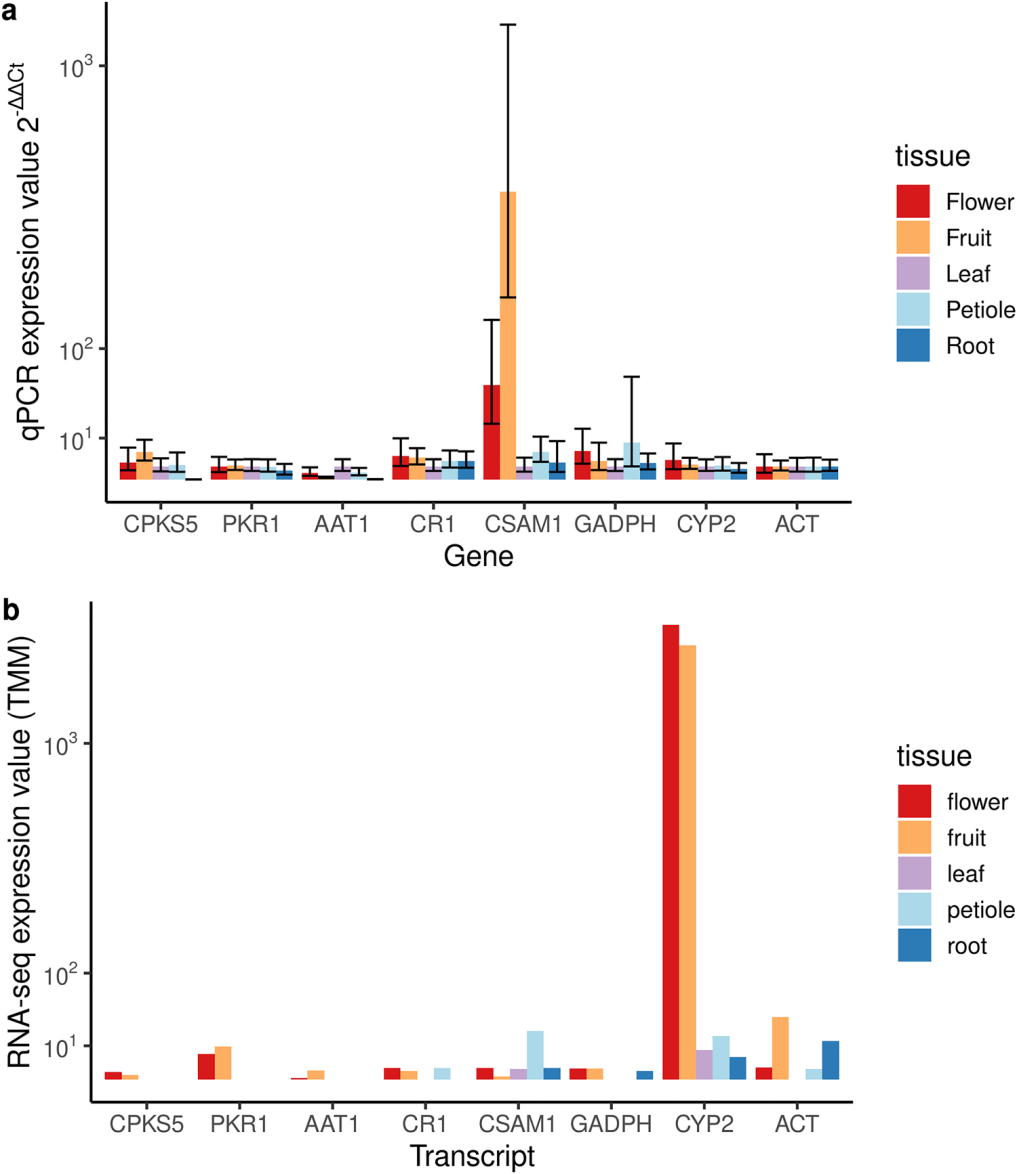
The expression profiling of selected gene candidates for *Conium* polyketide synthase 5 (CPKS5), polyketide reductase (PKR), l-alanine:5-keto-octanal aminotransferase (AAT), γ-coniceine reductase (CR), and *S*-adenosyl-l-methionine:coniine methyltransferase (CSAM) *in planta*. (**a**) The relative expression of CPKS5, PKR1, AAT1, CR1, and CSAM1 with housekeeping genes glyceraldehyde-3-phosphate dehydrogenase (GADPH) and cyclophilin 2 (CYP2). The relative expression is normalized using ACT and calculated using the 2^−ΔΔCt^-method and all values are compared to “leaf” within a plant (two biological replicates with three technical replicates, error bars are calculated using Eq. 1). (**b**) The number of the transcriptome reads for CPKS5, PKR1, AAT1, CR1, and CSAM1 with housekeeping genes ACT, GADPH and CYP2. The R package *ggplot* (https://ggplot2.tidyverse.org) was used to draw this figure.

We performed quantitative PCR experiments to confirm the expression patterns of selected genes observed as per RNA-seq data. For these validation experiments, we selected one gene candidate per enzyme of the coniine pathway together with three housekeeping genes (Supplementary Table S3). CPKS5 was expressed in the above-ground parts, i.e. stem, developing fruit, and flower but not in root (Fig. 6, Supplementary Fig. S8). Other candidates for the coniine pathway were expressed in flower and developing fruit similarly as CPKS5 and validated the patterns observed in the RNA-seq data.

## Discussion

Transcriptomes of nearly forty Apiaceae species are published (Supplementary Table S1). Here, we report the first transcriptome sequencing study of an Apiaceae species containing coniine, poison hemlock. Outside the Apiaceae family, transcriptomes of two *Sarracenia* species containing coniine^4^, *S. psittacina* and *S. purpurea*, have been published^32^. *Aloe* species containing coniine alkaloids remain to be sequenced.

Using de novo assembly of the transcriptomic reads, we constructed an assembly consisting of 123,240 transcripts. As the de novo assembly models the alternative splicing, obtaining many such transcripts is typical^23^. The assembly was functionally annotated using three complementary approaches—sequence alignment with the Uniprot-Swissprot database (using Trinotate^26^), PFAM protein family searches (using HMMER^28^), and the protein function classifier Pannzer2^25^—to increase the coverage of functional annotations. The combined use of all three tools increased the coverage of functional annotations to nearly 80% of the transcripts in the assembly. Separately, Trinotate, HMMER, and Pannzer2 annotated almost 12%, 38% and 50% of transcripts, respectively.

The quality of the transcriptome assembly was assessed for the evolutionarily-informed completeness of near-universal single-copy orthologs (BUSCO^33^) by comparing the transcriptome assembly with the ortholog data set of Eudicots clade (OrthoDB^34^). Over 88% of the near-universal orthologs of Eudicots clade were found in the transcriptome assembly. Selection of the most appropriate ortholog data set is crucial to interpret the results correctly. Eudicots constitute a large clade of flowering plants, including the Apiaceae family, where *C. maculatum* belongs. The Eudicots ortholog data set consists of single-copy orthologs that are expected to be present in at least 90% of the species in the clade. This information is based on 76 sequenced genomes of species belonging to various families of the clade. In particular, the ortholog data set includes only one Apiaceae species, *Daucus carota*.

On the other hand, to serve as a statistically meaningful near-universal ortholog data set, the data set needs to contain a significant number of genomes represented^34^. Given the diversity of the species included in the ortholog data set, the finding that more than 88% of orthologs are found complete in the *C. maculatum* transcriptome implies that the transcriptome assembly is of high quality in its gene content. BUSCO analysis has also deduced that nearly 15% of the orthologs are single-copy. In comparison, 73% were duplicated in the transcriptome assembly. This high duplication rate is typical when assessing de novo transcriptome assemblies as the assembly algorithm explicitly models alternative splicing and derives multiple isoforms for each gene^23^. In contrast, only a genome assembly can reveal the correct copy numbers of the universal orthologs^33^.

The overall gene expression patterns were studied in terms of the distributions of the numbers of genes expressed across the various organs (root, stem, leaf, flower, and developing fruit) of the poison hemlock (Fig. 3). To compare the expression values between the various organs, the data were between-sample normalised using TMM (Trimmed Mean of M-values)^35^. The transcript expression values in replicate samples of each organ were averaged to derive an organ-specific expression value for the transcripts. The ranked list of transcripts based on the expression values was then used for annotating each organ in terms of gene ontology biological terms associated with the genes expressed in the organ (Fig. 4, Supplementary Data S2). As there were only two replicate samples for each organ, no other statistical analysis was performed with the data. The expression patterns of the transcripts annotated with the plant hormone biosynthesis (KEGG pathway ID: *map01070*) are nevertheless provided as an example (Supplementary Fig. S3–S7). This paper, however, focuses on identifying the coniine biosynthesis pathway genes, as coniine is the most important secondary metabolite produced by *C. maculatum*.

Searching for coniine biosynthesis pathway genes, we found two candidates for CPKS5, two for PKR, six for AAT, and seven for CR and CSAM. CPKS5 has been enzymatically characterised earlier^9^. PKR involved in the pathway has not been described so far. We utilised kecG^20^, a KR domain, for in silico candidate selection. In plants, PKRs are rarely described in plant polyketide biosynthesis pathways. The most well-known is NADPH-dependent chalcone reductase in the flavonoid pathway^36^. Other known plant PKRs are tetraketide α-pyrone reductases in sporopollenin synthesis^37^, NADPH-dependent raspberry ketone/zingerone synthase 1 (RZS1) of *Rubus idaeus*^38^ and reductases participating in type I PKS complex in alkamide production in *Heliopsis longipes*^39^.

The *Conium* AAT has been characterised in enzymatic terms^10,11,24,40^. We utilised the molecular weight of the enzyme, the substrates, and the expression pattern of CPKS5 for the in silico selection of seven transcripts. Three of the seven transcripts shared the aminotransferase class-III protein family (PFAM PF00202) with kecF^20^. At the same time, one of them (TRINITY_DN92259_c1_g2_i1) was found by the sequence alignment search and protein family search. Unger^12^ described a spinach AAT (glutamate–oxaloacetate transferase), which formed coniine from l-alanine and 5-ketooctanal in in vitro testing. Based on his results, he concluded that coniine formation on spinach AAT is a side reaction for the enzyme. Together these two enzymes confirm that AAT is a non-specific alanine transferase, which donates nitrogen groups to multiple substrates with differing specificities. Therefore, a coniine-specific transamination reaction might be a side reaction for *C. maculatum* AAT. We applied broad criteria for selecting candidates to address this, and further testing is needed to confirm pathway-specific activity.

Following transamination, CR is the next putative enzyme in the pathway. We identified eight transcripts for the enzyme using kecH^20^ as a seed sequence, expression in flower and developing fruit like CPKS5, and utilisation of NADPH as a cofactor^13^. Plants contain many NAD(P)H-dependent reductases, such as a class II cytochrome P450 reductase^41^ and a functionally diverged short-chain dehydrogenase^42^ involved in alkaloid biosynthesis; the final candidate list also reflects this fact. Roberts^13^ postulates that either one enzyme performing two reactions (γ-coniceine + NADPH + H^+^ ↔ coniine + NADP^+^) (γ-coniceine reductase/coniine dehydrogenase), or two enzymes catalysing the reactions separately (γ-coniceine reductase: γ-coniceine + NADPH + H^+^ → coniine + NADP^+^; coniine dehydrogenase: coniine + NADP^+^ → γ-coniceine + NADPH + H^+^) could be involved. Many dehydrogenases catalyse the same reaction in both directions. Enzymatic tests show however that the enzyme would favour a reaction direction from γ-coniceine to coniine^13^. Therefore, it is likely that there is only one enzyme involved in the two reactions.

Roberts^14,15^ characterised an *N*-methyltransferase, using SAM as a methyl group donor. PFAM database contains multiple protein families of methyltransferases. The plant-specific methyltransferase family PF03492 acts on various substrates such as e.g. salicylic acid and alkaloid precursors. Due to this fact, we utilised this protein family for the in silico selection of CSAM candidates, followed by the enzyme classification into amine-*N*-methyltransferases (EC: 2.1.1.49). Generally, plants contain many well-known alkaloid *N*-methyltransferases. The first cloned representative is the 7-methylxanthine synthase of *Coffea arabica,* which methylates 7-methylxanthine to theobromine in caffeine biosynthesis^43^. Often members of this family function at the end of a route, but sometimes they initiate it. Interestingly, anthranilate *N*-methyltransferase initiates acridone biosynthesis before *N*-methylanthranilate is further processed by a PKS^44^.

We performed qPCR experiments for selected genes to validate their expression patterns obtained by RNA-seq data. We used second-year plants for qPCR because they have all the sequenced organs present to be comparable to RNA-seq. The validation experiments were done for selected coniine pathway candidates and housekeeping genes (Supplementary Table S3). The expression pattern of CPKS5 followed the observed RNA-seq data, i.e. CPKS5 was only expressed in plant parts above-ground (Fig. 6, Supplementary Fig. S8), correlating with the absence of coniine alkaloids in roots in the second year of growth^16,18^. The other candidate genes for the pathway were expressed in developing fruit and flower as expected. The expression pattern for CYP2 differs in qPCR and RNA-seq data for flower and developing fruit as it was quantified relative to actin in qPCR data. CSAM1 expression pattern was different in RNA-seq from that in qPCR, especially in developing fruit and flower, because (1) the aforementioned organs are still metabolically active as compared to e.g. leaves which was in senescence at the time of collecting samples and (2) the control sample for qPCR data is “leaf” to which all data is normalized. Roberts^19^ points out that CSAM is active in leaves when they are young. Observed disparities between RNA-seq and qPCR could either be due to the candidate genes having other functions or due to the different source material. Finally, a reference genome-based transcriptome assembly may provide more accurate expression estimates and a larger sample size would allow for better account of individual variance.

We acknowledge that in silico selection of enzyme candidates has some pitfalls. We have employed multiple sequence search approaches, functional annotation tools, and filtering criteria derived from our a priori knowledge of the coniine pathway to select the enzyme candidates as best as possible. For example, using the molecular weight alone, i.e. 56.12 kDa, as determined by Roberts^11^, for filtering during the selection of AAT (n = 302) retrieves TRINITY_DN92037_c0_g1_i3. However, TRINITY_DN92037_c0_g1_i3 is a 1-aminocyclopropane-1-carboxylate synthase-like gene based on functional annotations. Therefore, it is an unlikely candidate for AAT due to its different biological function. In other words, the predicted functional annotations do not support the involvement of this gene in alanine transamination reaction on coniine biosynthesis. Our data has shown that the CPKS5 was expressed in developing fruit and flower (Fig. 6). Thus, we used the expression in fruit and flower as a criterion for selecting candidates for the other pathway enzymes. We also assessed the utility of a more rigorous co-expression analysis of the transcripts across the five organs (Supplementary Fig. S9–S12). As demonstrated by the examples below, our data suggest that careful use of a priori knowledge combined with advanced bioinformatics tools for functional annotation, cofactor prediction, molecular weight determination, and sequence searches may be more valuable than co-expression analysis alone. For example, the AAT candidates TRINITY_DN94208_c2_g1_i1 and TRINITY_DN81865_c0_g1_i1 found based on sequence search and molecular weight criteria were also most highly correlated with CPKS5. However, they were predicted to be probable aminotransferase and aspartate aminotransferase, respectively, based on the functional annotations. On the other hand, TRINITY_DN92259_c1_g2_i1 was the AAT candidate least correlated with CPKS5 but was predicted to be alanine aminotransferase as required. Likewise, the CR candidate most highly correlated with CPKS5, TRINITY_DN101573_c2_g1_i10, does not utilize NADPH as the cofactor, making it an unlikely candidate for CR. The co-expression analysis alone may be more valuable when we lack the knowledge of the studied pathway and when large amounts of expression data are available.

In conclusion, we provide the first report for transcriptome sequencing of poison hemlock. The transcriptome assembly, containing over 88% of the near-universal orthologs of Eudicots clade, is of good quality. This study further proposes in silico candidates for PKR, AAT, CR, and CSAM in the coniine biosynthesis pathway. In vitro testing is needed to further confirm the selected candidates' functions, followed by *in planta* confirmation. Whether or not coniine biosynthesis uses more enzymes is still open. However, we have not found any candidates similar to, *e*.*g*. polyketide cyclase. Sequencing the genome of poison hemlock, e.g. using long-read technologies such as PacBio, would complement the current RNA-seq data and provide further insights into the coniine biosynthesis.

## Materials and methods

### Plant material

Root, stem, and leaf samples of poison hemlock (*Conium maculatum* L.) were collected from a greenhouse-grown second-year plant during the 2011 winter. The plant was grown from seeds collected in Helsinki, Finland (60.238482°N, 25.033406°E). The controlled greenhouse conditions were: temperature 20 °C, humidity 60%, and a photoperiod of 16 h:8 h, light:dark. The potting soil was half vermiculite and half peat (Kekkilä Oy, Finland). Flower and developing fruit were collected in July 2011 in Helsinki, Finland (60.214250°N, 24.917459°E). Two whole second-year plants for qPCR testing were collected in July 2022 in Helsinki, Finland from the same location as the seeds for greenhouse grown plants.

### RNA extraction

Total RNA was isolated from root, stem, leaf, flower, and fruit of poison hemlock in two replicates with the pine tree method^45^. The quality and quantity of RNA was checked with a NanoDrop 2000 (Thermo Scientific, Wilmington, DE, USA). Genomic DNA was removed with DNase (RNase free, Fermentas, Leon-Rot, Germany).

### Transcriptome sequencing

The RNA quality was measured with an Agilent 2100 Bioanalyzer, and the mRNA fraction was paired-end sequenced by the Illumina SOLiD platform at Biomedicum Functional Genomics Unit (Helsinki, Finland). The sequence read data were processed as presented in Fig. 2. The quality of the raw sequencing reads was reported as Phred 64 scores, as it was analysed using FASTQC (v0.11.8). Quality filtering and trimming were done using Trimmomatic (v0.35).

### Assembly of the transcriptome

The quality-filtered paired-end reads of all organs (root, stem, leaf, flower, and developing fruit) were pooled and assembled de novo (i.e. without any reference genome sequence) using Trinity^23^. The BUSCO approach^33^ was used to evaluate the evolutionarily-expected completeness of the transcriptome assembly based on the presence of Universal Single-Copy Ortholog genes. The Eudicots clade level ortholog data set from OrthoDB v 10^34^ was used in the BUSCO (v 5.1.3) analysis, consisting of the set of orthologous genes expected to be present in over 90% of the species in the taxonomic clade of Eudicots.

### Annotation of the transcriptome

Gene prediction (prediction of open reading frames, ORFs) and the translation of coding sequences to peptides were made using Trinotate^23^. Functional annotations of the coding sequences were performed by using BLAST^46^ with the UniProt-SwissProt database^27^ (using Trinotate), hidden Markov model (HMM) search with protein family (PFAM) database^47^, and Pannzer2^25^. The BLAST annotations were filtered using the minimum identity percentage threshold of 80 and the minimum alignment coverage threshold of 50% of the amino acid sequence. After this filtering, the BLAST search results had an E-value less than 10^−20^. Therefore, the maximum E-value threshold of 10^−20^ was chosen to filter the HMM search results. The Pannzer results were first screened by using a minimum positive predictive value (PPV) threshold of 0.5, determined heuristically, and secondly by retaining only those annotations whose PPV score was ≥ 0.9 × max(PPV) for each ORF. By retrieving a mapping between Uniprot identifiers and enzyme commission (EC) numbers using the Uniprot REST API, Trinotate-based functional annotations were linked to enzymes. The fully determined enzyme class annotations derived from this mapping and Pannzer were linked to KEGG metabolic pathways using the KEGG REST API^29^. The mapped enzyme annotations were visualized as the global metabolic pathway network (KEGG ID: map01100) and the network of biosynthesis of secondary metabolites (KEGG ID: map01110) using iPath 3.0^48^.

The transcriptome assembly was decontaminated by removing the transcripts whose sequences are not likely originating from Plantae. The transcripts annotated exclusively with genes belonging to Bacteria or Opisthokonta (i.e. fungi and Animalia) based on the UniProt annotations derived by Trinotate were removed.

### Quantitative PCR (qPCR)

Total RNA isolated from leaf, root, stem, developing fruit, and flower were synthesized into cDNA using QuantiTect Reverse Transcription Kit (QiaGEN, Hilden, Germany) according to the manufacturer’s instructions. The sequences for CPKS5 (TRINITY_DN93485_c1_g2_i1 and TRINITY_DN93485_c1_g2_i6), PKR1 (TRINITY_DN97220_c2_g4_i5), AAT1 (TRINITY_DN100028_c0_g1_i9), CR1 (TRINITY_DN86784_c0_g1_i2), CSAM1 (TRINITY_DN100232_c1_g3_i3), GADPH (TRINITY_DN96139_c0_g2_i5), CYP2 (TRINITY_DN93680_c4_g1_i1), and ACT (TRINITY_DN98945_c6_g1_i1) were obtained via transcriptome sequencing and the primers for qPCR were designed using PrimerQuest software (https://eu.idtdna.com/PrimerQuest/Home/Index). The primers (Supplementary Table S3) were ordered from IDT (Leuven, the Netherlands). The qPCR machine was a LightCycler 480 II (Roche Diagnostics Ltd, Rotkreuz, Switzerland) and white 96-well plates were used. The efficiency of the primers was tested using genomic DNA as template which was isolated using the CTAB method^9^. The volume of the reaction mixture was 20 µl which contained 10 µl 2 × master mix (LightCycler 480 SYBR Green I Master, version 13, Roche Diagnostics GmbH, Mannheim, Germany), 2.5 µl 8 µM primer stock containing both forward and reverse primers, and 12.5 ng cDNA as template. Each experiment was done in triplicate. The temperature program was as follows: pre-incubation 95 °C for 5 min; amplification for 45 rounds 95 °C for 10 s, 60 °C for 10 s and 72 °C for 10 s; and melting curve 95 °C for 5 s, 65 °C for 1 min and 97 °C continuous. The relative transcriptional changes in gene expression levels (fold changes) were calculated using the comparative C_t_-method (2^−ΔΔCt^)^49^ using the housekeeping genes glyceraldehyde-3-phosphate dehydrogenase (GADPH), actin (ACT) and cyclophilin 2 (CYP2) as a reference. The standard deviation for ΔΔC_t_ was calculated using the propagation of error method according to this formula:

1 $$\sigma \Delta \Delta C_{tGOI} = \sqrt {\left( {\sigma C_{tGOI} } \right)_{leaf}^{2} + \left( {\sigma C_{tHKG} } \right)_{leaf}^{2} + \left( {\sigma C_{tGOI} } \right)_{organ}^{2} + \left( {\sigma C_{tHKG} } \right)_{organ}^{2} }$$

### Biological pathways expressed in each organ

The gene ontology (GO)^50^ biological process terms enriched among the genes expressed in each organ were found by using gene set enrichment analysis (GSEA)^30^. The gene expression values were estimated using FPKM (Fragments per kilobase per million mapped reads) and between-sample using TMM implemented in edgeR R package^35^. The TMM expression values of genes expressed in each organ (root, stem, leaf, flower, and developing fruit), averaged between the replicates, were used to rank the genes from the most highly expressed to the least expressed gene. They were used in the GSEA analysis of ranked lists, and the GO annotations derived during the annotation of the transcriptome were used as the gene set database. When creating the ranked lists of genes expressed in each organ, the genes that are not expressed in an organ were excluded from the data for the organ. A relaxed statistical significance level of 1% (nominal *p* value of < 0.01) was set to get a description of each organ with at least several GO terms because the transcriptome data contain multiple isoforms for each gene, which are likely to highly correlate with each other, and the objective of GSEA was to understand the gene expression patterns of each organ in terms of the gene ontology biological process annotations.

### Identification of coniine biosynthesis pathway enzymes

#### Identification of CPKS5 candidates

To identify the transcript candidates encoding the CPKS5 gene, a protein–protein BLAST search was performed with the protein sequence obtained from Uniprot (accession: A0A0K0TQH1) as the query sequence and the peptide sequences predicted from the transcriptome assembly as the subject sequence database. The transcript candidates for CPKS5 were found from the homology matches in the transcriptome assembly using the combination of high percentage identity and sequence length coverage in the alignment.

#### Identification of aminotransferases

To identify the aminotransferases, a combination of the following sequence homology searches was applied:

- Homology search using BLAST (filtered by using the identity ≥ 80% and alignment length ≥ 50% of the query amino acid sequences) against all transaminase sequences collected from the Uniprot database (the transaminase sequences used for the search are provided in Supplementary Data S4)
- Homology search using BLAST (filtered by using the identity ≥ 80% and alignment length ≥ 50% of the query amino acid sequences) against all transaminase sequences from protein data bank (PDB) (the transaminase sequences used for the search are provided in Supplementary Data S4), and
- HMM search for the PFAM protein families PF00155 (Aminotransferase class I and II), PF00202 (Aminotransferase class III), PF00266 (Aminotransferase, class V) (filtered with domain E-value < 10^−20^, as explained in the Annotation of the transcriptome section).

The union of the search results obtained by all three approaches was taken as the initial list of candidates to facilitate the identification of the alanine aminotransferase (AAT) involved in the coniine biosynthesis. The list was filtered based on the co-expression with CPKS5 candidates. Roberts^11^ determined the molecular weight of AAT as 56.2 kDa. This molecular weight was used to filter the preliminary candidates for AAT obtained through sequence searches. To this end, the molecular weights of the protein sequences predicted from the transcripts in the *C. maculatum* transcriptome were calculated using the R package Peptides^51^. To account for possible uncertainty in the molecular weight, which may arise from the fact that the molecular weights of the *C. maculatum* proteins have been computationally predicted based on the translated sequences alone or that the exact molecular weight may also be affected by post-translational modifications, a molecular weight-range around the experimentally determined value^11^ was used. The sequence homology search results were filtered with the 45–65 kDa molecular weight range. Finally, using the functional annotations derived from our annotation pipeline as additional information, the most probable candidates for AAT in the coniine biosynthesis pathway were manually selected by removing the aminotransferases that do not use alanine as substrate.

#### Identification of enzyme candidates for PKR, CR, and CSAM

To find the *C. maculatum* transcript candidates encoding the polyketide and γ-coniceine reductases in the coniine biosynthesis, the protein family domain signatures of *P. koreensis* gene sequences in the koreenceine biosynthetic pathway were taken as the seeds to search the poison hemlock transcriptome. To this end, the *C. maculatum* assembly and the *P. koreensis* genes were annotated by protein family domain signatures of PFAM-database using the hidden Markov models (HMMER) with a maximum domain E-value threshold of 10^−20^ (see the Annotation of the transcriptome section). The sequences were matched between the species based on the shared PFAM domains. Based on in vitro tests^13^, CR is an NADPH-dependent enzyme. Therefore, to select the most probable candidates, cofactor utilisation was predicted from the sequences using Cofactory^52^. The sequences that were predicted to utilise NADPH, NADPH/NADH, or NADH as cofactors were first selected. EC-classification was used further to select CR candidates (EC = 1.3.1, oxidoreductase acting on the CH-CH group of donors with NAD^+^, NADP^+^ as acceptors)*.* The CSAM enzyme reaction requires methylation of the nitrogen group of the substrate, and, based on in vitro testing, the donor is SAM^14,15,53^. Therefore, to identify CSAM candidates, the transcripts were first screened for the SAM binding domain (PFAM 03492, SAM-dependent carboxyl methyltransferase). They were further filtered for E.C. 2.1.1.49 (amine-*N*-methyltransferases). The co-expression with CPKS5 was an additional criterion for selecting all enzyme candidates.

### Sample collection

According to the Finnish law 769/1990 section 28 § 14 (Jokamiehen oikeudet/Every man’s rights) the collection of above ground parts of an unprotected plant is legal without a permission. Poison hemlock (*Conium maculatum*) is not protected by the Finnish law. The voucher specimen (H855954) is deposited at the Finnish Museum of Natural History, Botanical Museum (H) and it was identified by Hannu Hotti.

## Supplementary Information

Supplementary Information.

## Data Availability

All the essential data associated with this manuscript are made available as supplementary data with an open access license (CC BY 4.0) at the web addresses mentioned above. The sequencing raw data are available from European Nucleotide Archive with the project accession number PRJEB56429.
