# Supplementary material for: De novo transcriptome assembly of Conium maculatum L. to identify candidate genes for coniine biosynthesis
Source: Sci Rep. 2022 Oct 20;12:17562. doi: 10.1038/s41598-022-21728-w (PMC9584964; doi:10.1038/s41598-022-21728-w)
Supplement: Supplementary file 1 — Supplementary Information. [file 41598_2022_21728_MOESM1_ESM.pdf]

Supplementary material to: *De novo* transcriptome assembly of *Conium maculatum L.* to identify candidate genes for coniine biosynthesis

Gopal Peddinti[1†], Hannu Hotti[1†¶], Teemu H. Teeri[2] and Heiko Rischer[1]

[1]VTT Technical Research Centre of Finland Ltd, Tietotie 2, Espoo, P.O. Box 1000, FI-02044 VTT, Finland

[2]Viikki Plant Science Centre, Department of Agricultural Sciences, University of Helsinki, PO Box 27, FI-00014 Helsinki, Finland

[†]These authors contributed equally to this work.

[¶]Present address: Faculty of Biological and Environmental Sciences, University of Helsinki, PO Box 56, FI-00014 Helsinki, Finland

Supplementary Table S1

Available online as a word document via https://doi.org/10.6084/m9.figshare.17372195

Sequenced transcriptomes of Apiaceae.

**Genus**	**Species**	**Common name**	**Organ(s)**	**Next-generation sequencing method**	**Reference**
Aegopodium	*podagraria*	Ground elder	Leaves	Illumina NovaSeq 6000	[1]
Angelica	*acutiloba*	Toki	Leaves	HiSeq X Ten	[1]
Angelica	*decursiva*		Leaves	HiSeq X Ten	[1]
Anthriscus	*sylvestris*	Cow parsley	Leaves	Illumina NovaSeq 6000	[1]
Apium	*graveolens*	Celery	Leaves	Illumina HiSeq 2000	[2]
Bupleurum	*chinense*		Leaves	HiSeq X Ten	[1]
Centella	*asiatica*	Indian pennywort	Leaves	Illumina Genome Analyzer II	[3]
Chamaesium	*paradoxum*		Leaves	Illumina NovaSeq 6000	[1]
Cnidium	*monnieri*	Monnier's snowparsley	Leaves	Illumina NovaSeq 6000	[1]
Coriandrum	*sativum*	Coriander	different developmental stages of mericarps (seeds)	Illumina GAIIx	[4]
			Leaves	Illumina NovaSeq 6000	[1]
Cryptotaenia	*japonica*	Mitsuba	Leaves, roots	Illumina HiSeq 2000	[5]
			Leaves	Illumina NovaSeq 6000	[1]
Cyclospermum	*leptophyllum*	Marsh parsley	Leaves	Illumina NovaSeq 6000	[1]
Daucus	*carota*	Carrot	The whole plant (14 different accessions)	Illumina HiSeq 2000	[6]

Ferula	*assafoetida*	Asafoetida	Root, stem, leaves, flowers	Illumina HiSeq 4000	[7]
Ferula	*gummosa*	Galbanum	Roots, flowers	Illumina HiSeq 2000	[8]
Foeniculum	*vulgare*	Fennel	Leaves	Illumina NovaSeq 6000	[1]
Haplosphaera	*phaea*		Leaves	Illumina NovaSeq 6000	[1]
Heracleum	*candicans*		Leaves	HiSeq X Ten	[1]
Hymenidium	*davidii*		Leaves	Illumina NovaSeq 6000	[1]
Ligustum	*chuanxiong*		Leaves, root	Illumina HiSeq 2500	[9]
Ligusticum	*jeholense*		Leaves	Illumina NovaSeq 6000	[1]
Nothosmyrnium	*japonicum*		Leaves	HiSeq X Ten	[1]
Notopterygium	*franchetii*		Leaves, stems, flowers (mixed sample)	Illumina HiSeq 2000	[10]
Notopterygium	*incisum*	Notopterygium root	Leaves, stems, flowers (mixed sample)	Illumina HiSeq 2000	[10]
Oenanthe	*javanica*	Java water dropwort	Leaves	Illumina NovaSeq 6000	[1]
Oenanthe	*thomsonii*	Thomson's water dropwort	Leaves	Illumina NovaSeq 6000	[1]
Ostericum	*grosseserratum*	Danggwi	Leaves	Illumina NovaSeq 6000	[1]
Pastinaca	*sativa*	Parsnip	Leaves	Illumina NovaSeq 6000	[1]
Petroselinum	*crispum*	Parsley	Leaves, stems, root (mixed sample)	Illumina HiSeq 2000	[11]
Peucedanum	*japonicum*	Coastal hog fennel	Leaves	Illumina NovaSeq 6000	[1]
Pimpinella	*diversifolia*		Leaves	Illumina NovaSeq 6000	[1]
Pternopetalum	*trichomanifolium*		Leaves	Illumina NovaSeq 6000	[1]
Pternopetalum	*vulgare*		Leaves	Illumina NovaSeq 6000	[1]
Sanicula	*orthacantha* var. *stolonifera*		Leaves	Illumina NovaSeq 6000	[1]

Saposhnikovia	*divaricata*	Siler	Leaves	Illumina NovaSeq 6000	[1]
Thapsia	*laciniata*	Villous deadly carrot	root	Illumina GAIIx	[12]
Torilis	*scabra*	Rough hedge parsley	Leaves	Illumina NovaSeq 6000	[1]
Trachyspermum	*ammi*	Ajowan	Inflorescens	Illumina HiSeq 2000	[13]

Supplementary Table S2

Available online via https://doi.org/10.6084/m9.figshare.19778815

The list of KEGG metabolic pathways[14] and the distribution of EC number annotations of *Conium maculatum* L. among them.

KEGG Pathway id	Pathway name	EC found in assembly	Number of EC in KEGG reference pathway	Percent EC in assembly
map00195	Photosynthesis	3	3	100.00
map00511	Other glycan degradation	8	9	88.89
map00710	Carbon fixation in photosynthetic organisms	22	25	88.00
map00592	alpha-Linolenic acid metabolism	11	13	84.62
map01040	Biosynthesis of unsaturated fatty acids	11	14	78.57
map00062	Fatty acid elongation	10	13	76.92
map00061	Fatty acid biosynthesis	13	17	76.47
map00970	Aminoacyl-tRNA biosynthesis	23	31	74.19
map00020	Citrate cycle (TCA cycle)	18	26	69.23
map00983	Drug metabolism - other enzymes	18	26	69.23
map00073	Cutin, suberine and wax biosynthesis	6	9	66.67
map00563	Glycosylphosphatidylinositol (GPI)-anchor biosynthesis	2	3	66.67
map00290	Valine, leucine and isoleucine biosynthesis	9	14	64.29
map00010	Glycolysis / Gluconeogenesis	32	50	64.00
map00982	Drug metabolism - cytochrome P450	5	8	62.50
map00100	Steroid biosynthesis	18	29	62.07
map00220	Arginine biosynthesis	19	31	61.29
map00190	Oxidative phosphorylation	6	10	60.00
map00250	Alanine, aspartate and glutamate metabolism	29	50	58.00
map00400	Phenylalanine, tyrosine and tryptophan biosynthesis	22	38	57.89
map00562	Inositol phosphate metabolism	26	48	54.17
map00564	Glycerophospholipid metabolism	34	63	53.97
map00940	Phenylpropanoid biosynthesis	14	26	53.85

map00941	Flavonoid biosynthesis	15	28	53.57
map00450	Selenocompound metabolism	9	17	52.94
map00900	Terpenoid backbone biosynthesis	27	51	52.94
map00260	Glycine, serine and threonine metabolism	37	70	52.86
map00600	Sphingolipid metabolism	17	33	51.52
map00770	Pantothenate and CoA biosynthesis	17	33	51.52
map00230	Purine metabolism	55	108	50.93
map00261	Monobactam biosynthesis	5	10	50.00
map00280	Valine, leucine and isoleucine degradation	18	36	50.00
map00513	Various types of N-glycan biosynthesis	12	24	50.00
map00905	Brassinosteroid biosynthesis	2	4	50.00
map00980	Metabolism of xenobiotics by cytochrome P450	5	10	50.00
map00561	Glycerolipid metabolism	22	45	48.89
map00480	Glutathione metabolism	19	39	48.72
map00510	N-Glycan biosynthesis	17	35	48.57
map00670	One carbon pool by folate	13	27	48.15
map00730	Thiamine metabolism	13	28	46.43
map00270	Cysteine and methionine metabolism	39	86	45.35
map00240	Pyrimidine metabolism	27	62	43.55
map00565	Ether lipid metabolism	10	23	43.48
map00790	Folate biosynthesis	23	53	43.40
map00620	Pyruvate metabolism	32	74	43.24
map00130	Ubiquinone and other terpenoid-quinone biosynthesis	19	45	42.22
map00945	Stilbenoid, diarylheptanoid and gingerol biosynthesis	5	12	41.67
map00281	Geraniol degradation	4	10	40.00
map00332	Carbapenem biosynthesis	2	5	40.00
map00785	Lipoic acid metabolism	2	5	40.00
map00908	Zeatin biosynthesis	4	10	40.00
map00052	Galactose metabolism	19	48	39.58
map00500	Starch and sucrose metabolism	30	76	39.47

map00910	Nitrogen metabolism	15	39	38.46
map00780	Biotin metabolism	8	21	38.10
map00460	Cyanoamino acid metabolism	11	29	37.93
map00740	Riboflavin metabolism	11	29	37.93
map00410	beta-Alanine metabolism	14	37	37.84
map00300	Lysine biosynthesis	12	33	36.36
map00071	Fatty acid degradation	11	31	35.48
map00720	Carbon fixation pathways in prokaryotes	18	51	35.29
map00760	Nicotinate and nicotinamide metabolism	21	60	35.00
map00520	Amino sugar and nucleotide sugar metabolism	44	127	34.65
map00030	Pentose phosphate pathway	19	55	34.55
map00053	Ascorbate and aldarate metabolism	20	58	34.48
map00630	Glyoxylate and dicarboxylate metabolism	26	76	34.21
map00380	Tryptophan metabolism	21	62	33.87
map00340	Histidine metabolism	13	39	33.33
map00531	Glycosaminoglycan degradation	5	15	33.33
map00965	Betalain biosynthesis	1	3	33.33
map00051	Fructose and mannose metabolism	25	76	32.89
map01110	Biosynthesis of secondary metabolites	434	1321	32.85
map00643	Styrene degradation	6	19	31.58
map00591	Linoleic acid metabolism	4	13	30.77
map00350	Tyrosine metabolism	20	66	30.30
map00860	Porphyrin metabolism	32	107	29.91
map01100	Metabolic pathways	814	2756	29.54
map00514	Other types of O-glycan biosynthesis	4	14	28.57
map00960	Tropane, piperidine and pyridine alkaloid biosynthesis	8	28	28.57
map00750	Vitamin B6 metabolism	8	29	27.59
map00640	Propanoate metabolism	14	51	27.45
map00603	Glycosphingolipid biosynthesis - globo and isoglobo series	3	11	27.27
map00650	Butanoate metabolism	16	61	26.23

map00040	Pentose and glucuronate interconversions	19	73	26.03
map00920	Sulfur metabolism	14	54	25.93
map00330	Arginine and proline metabolism	22	85	25.88
map00360	Phenylalanine metabolism	15	58	25.86
map00310	Lysine degradation	18	71	25.35
map00401	Novobiocin biosynthesis	4	16	25.00
map00604	Glycosphingolipid biosynthesis - ganglio series	2	8	25.00
map00906	Carotenoid biosynthesis	11	44	25.00
map00590	Arachidonic acid metabolism	6	26	23.08
map00830	Retinol metabolism	3	13	23.08
map00521	Streptomycin biosynthesis	4	18	22.22
map00680	Methane metabolism	20	91	21.98
map00660	C5-Branched dibasic acid metabolism	5	23	21.74
map00950	Isoquinoline alkaloid biosynthesis	14	65	21.54
map01120	Microbial metabolism in diverse environments	155	720	21.53
map01051	Biosynthesis of ansamycins	1	5	20.00
map00944	Flavone and flavonol biosynthesis	6	32	18.75
map00930	Caprolactam degradation	4	22	18.18
map00943	Isoflavonoid biosynthesis	4	22	18.18
map00470	D-Amino acid metabolism	9	51	17.65
map00642	Ethylbenzene degradation	1	6	16.67
map00541	O-Antigen nucleotide sugar biosynthesis	10	63	15.87
map00430	Taurine and hypotaurine metabolism	3	19	15.79
map00901	Indole alkaloid biosynthesis	5	32	15.63
map00232	Caffeine metabolism	2	13	15.38
map00633	Nitrotoluene degradation	1	7	14.29
map00601	Glycosphingolipid biosynthesis - lacto and neolacto series	2	15	13.33
map00966	Glucosinolate biosynthesis	2	15	13.33
map00440	Phosphonate and phosphinate metabolism	4	31	12.90
map00402	Benzoxazinoid biosynthesis	1	8	12.50

map00512	Mucin type O-glycan biosynthesis	1	8	12.50
map00903	Limonene and pinene degradation	2	16	12.50
map00942	Anthocyanin biosynthesis	2	16	12.50
map00625	Chloroalkane and chloroalkene degradation	2	17	11.76
map00626	Naphthalene degradation	1	9	11.11
map00981	Insect hormone biosynthesis	1	9	11.11
map00904	Diterpenoid biosynthesis	7	65	10.77
map00552	Teichoic acid biosynthesis	2	20	10.00
map00902	Monoterpenoid biosynthesis	5	50	10.00
map00999	Biosynthesis of various plant secondary metabolites	4	43	9.30
map00791	Atrazine degradation	1	11	9.09
map00909	Sesquiterpenoid and triterpenoid biosynthesis	7	80	8.75
map00550	Peptidoglycan biosynthesis	2	23	8.70
map00362	Benzoate degradation	6	70	8.57
map01053	Biosynthesis of siderophore group nonribosomal peptides	1	12	8.33
map00627	Aminobenzoate degradation	4	51	7.84
map00254	Aflatoxin biosynthesis	1	13	7.69
map00405	Phenazine biosynthesis	1	13	7.69
map00311	Penicillin and cephalosporin biosynthesis	1	14	7.14
map00364	Fluorobenzoate degradation	1	14	7.14
map00140	Steroid hormone biosynthesis	2	36	5.56
map00524	Neomycin, kanamycin and gentamicin biosynthesis	1	28	3.57
map00998	Biosynthesis of various antibiotics	1	44	2.27

Supplementary Table S3

Available online as a word document via https://doi.org/10.6084/m9.figshare.21154894

The primers used in quantitative PCR. *Conium* polyketide synthase 5 (CPKS5), polyketide reductase (PKR), L-alanine:5-keto-octanal aminotransferase (AAT), γ-coniceine reductase (CR), and *S*-adenosyl-L-methionine:coniine methyltransferase (CSAM), glyceraldehyde-3-phosphate dehydrogenase (GADPH), cyclophilin 2 (CYP2), and actin (ACT).

Name		Sequence	Efficiency
CPKS5	F	CAGATTTCTGGAACATGGCAC	91,16%
	R	CTTGGTAAGCCGAAAGTCC	
PKR1	F	TTGTTCATATCGCCACTATCAC	92,93%
	R	ACG GGA GAG AGA TAC GAG AAA	
AAT1	F	ACTGAGGAAGAGAGCCCATA	94,04%
	R	TCAACTGCTTTCGGAGGTAAA	
CR1	F	TTAACTGGAGGTGGATCAGG	90,81%
	R	CGAGGGAGGTAAGAGAAGAAA	
CSAM1	F	GCTGAGGGTTTACTTCACAAGG	92,58%
	R	AAATGTGCCACTGCTATCC	
GADPH	F	GGCATTGTTGAGGGTCTTATG	108,7%
	R	AGTGCTGCTGGGAATGATA	
CYP2	F	CGAAGTTTGCTGACGAGAAT	97,2%
	R	CACAACATGCTTTCCATCCA	
ACT	F	GGCTTTGCTGGTGATGATGC	101,51%
	R	TTTCTGTCCCATTCCGACC	

Supplementary Figure S1

Available online via https://doi.org/10.6084/m9.figshare.19761646.

The KEGG (Kyoto Encyclopedia of Genes and Genomes[14]) metabolic network (KEGG pathway ID: *map01100 metabolic pathways*) with the reactions found in the transcriptome annotations highlighted in red based on the enzyme commission (EC) number annotations. Out of the 2756 EC numbers represented in the reference KEGG network, 814 were found in the transcriptome assembly. Figure was drawn with iPath3 software[15].

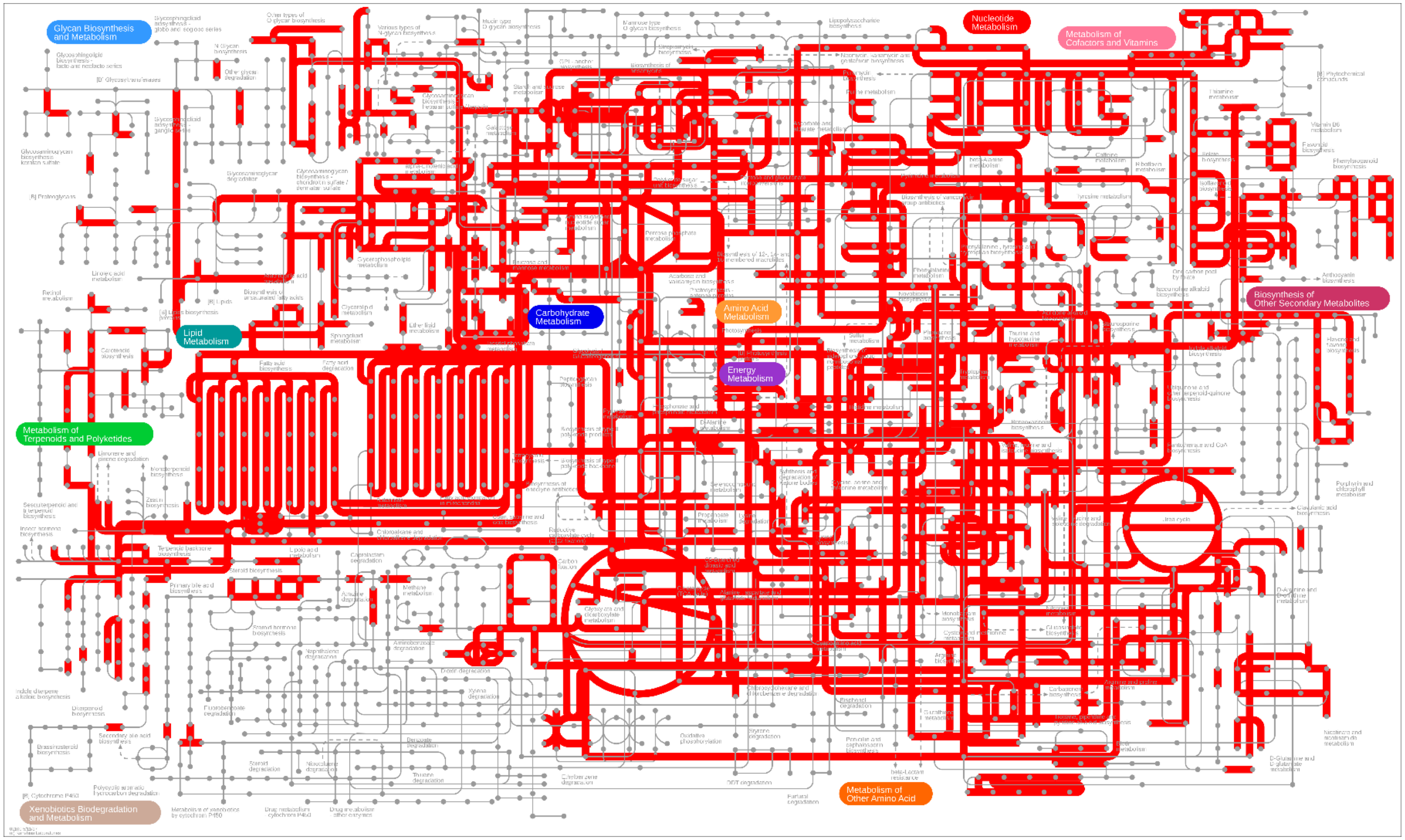

Supplementary Figure S2

Available online via https://doi.org/10.6084/m9.figshare.19761742.

The KEGG[14] network of biosynthesis of secondary metabolites (KEGG pathway ID: *map01110*) with the reactions found in the transcriptome annotations highlighted in red based on the enzyme commission (EC) number annotations. Out of the 1321 EC numbers represented in the reference KEGG network, 434 were found in the transcriptome assembly. The figure was drawn with iPath3 software[15].

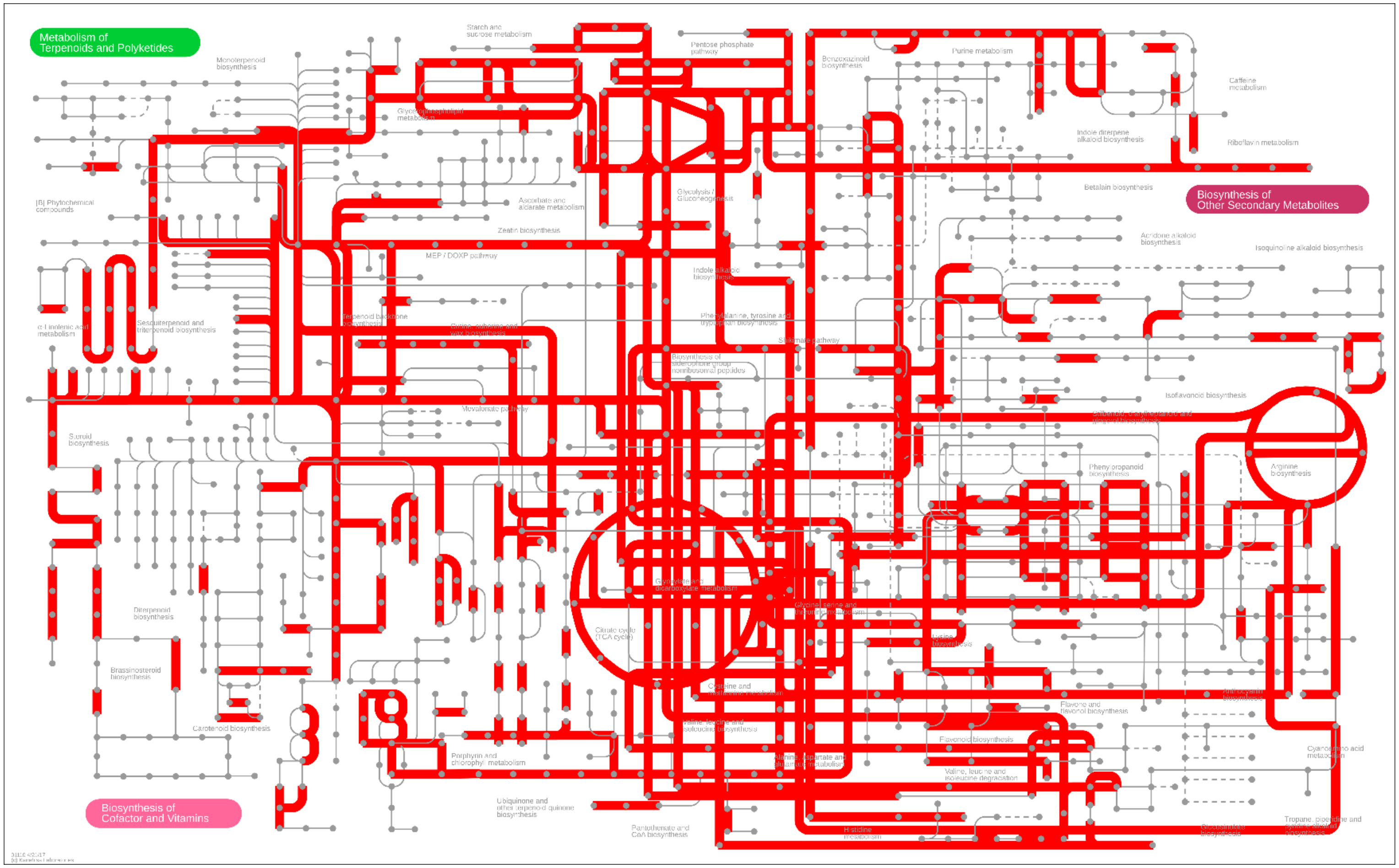

Supplementary Figure S3

Available online via https://doi.org/10.6084/m9.figshare.19782820 (NOTE: Because the image height exceeds the page height of this document, a rotated view of the figure is included below to minimize the loss of image quality. For better image quality, please check the online version).

The KEGG pathway map of the plant hormone biosynthesis consists of several sub-pathways. This figure shows the list and the expression patterns of transcripts in the Conium maculatum L. transcriptome assembly annotated to encode the enzymes of the fatty acid biosynthesis (KEGG ID path:map00061) of the plant hormone biosynthesis.

The expression values are shown in the square-root scale. The false discovery rate (FDR) q-values of the differential expression (between the organ indicated vs the rest of the organs) are shown with the "+" sign whenever Q-value < 0.1. Note that the differential expresison statistics could not be calculated for most of the transcripts due to the lack of sample size (two replicates). The figure was created with *pheatmap* R package.

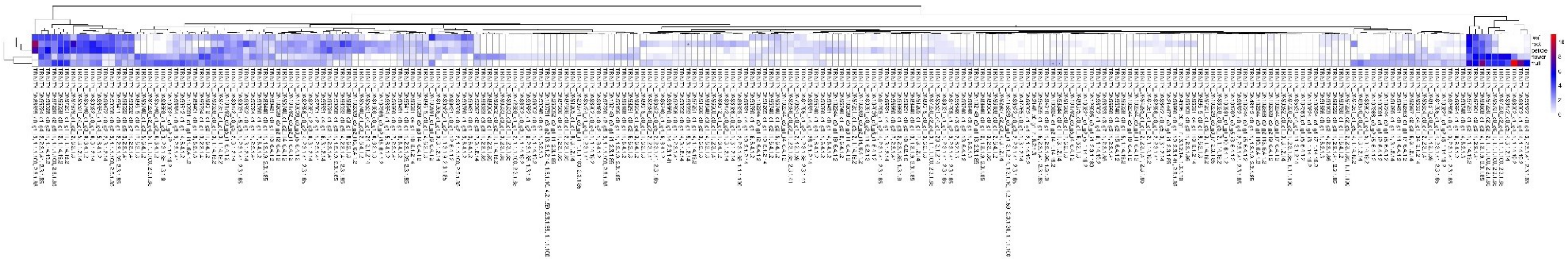

Supplementary Figure S4

Available online via https://doi.org/10.6084/m9.figshare.19782964 (NOTE: Because the image height exceeds the page height of this document, a rotated view of the figure is included below to minimize the loss of image quality. For better image quality, please check the online version).

The KEGG pathway map of the plant hormone biosynthesis consists of several sub-pathways. This figure shows the list and the expression patterns of transcripts in the *Conium maculatum* L. transcriptome assembly annotated to encode the enzymes of the terpenoid backbone biosynthesis (KEGG ID path:map00900) of the plant hormone biosynthesis.

The expression values are shown in the square-root scale. The false discovery rate (FDR) q-values of the differential expression (between the organ indicated vs the rest of the organs) are shown with the "+" sign whenever Q-value < 0.1. Note that the differential expression statistics could not be calculated for most of the transcripts due to the lack of sample size (two replicates). The figure was created with *pheatmap* R package.

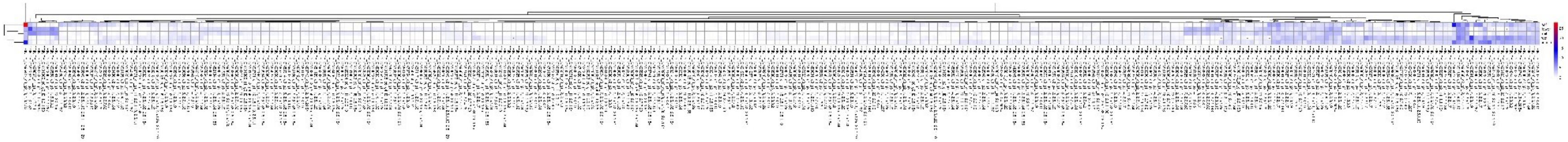

Supplementary Figure S5

Available online via https://doi.org/10.6084/m9.figshare.19783021

The KEGG pathway map of the plant hormone biosynthesis consists of several sub-pathways. This figure shows the list and the expression patterns of transcripts in the *Conium maculatum* L. transcriptome assembly annotated to encode the enzymes of the Brassinosteroid biosynthesis(KEGG ID path:map00905) of the plant hormone biosynthesis.

The expression values are shown in the square-root scale. The false discovery rate (FDR) q-values of the differential expression (between the organ indicated vs the rest of the organs) are shown with the "+" sign whenever Q-value < 0.1. Note that the differential expression statistics could not be calculated for most of the transcripts due to the lack of sample size (two replicates). The figure was created with *pheatmap* R package.

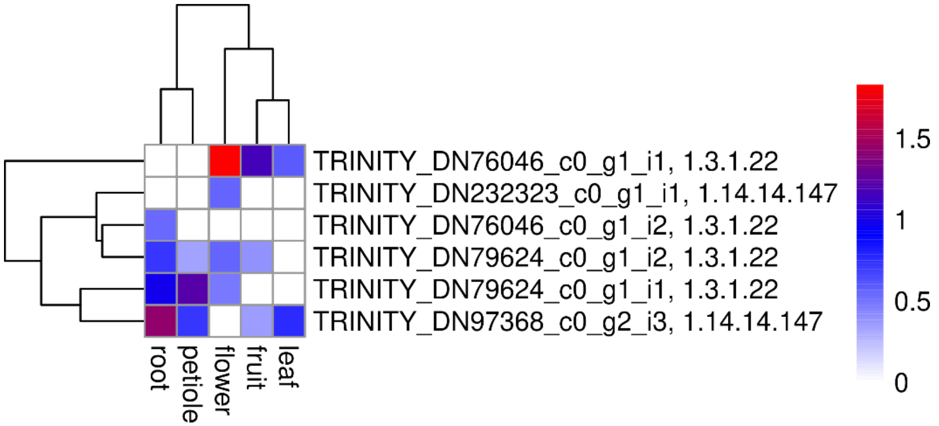

Supplementary Figure S6

Available online via https://doi.org/10.6084/m9.figshare.19783042 (NOTE: Because the image height exceeds the page height of this document, a rotated view of the figure is included below to minimize the loss of image quality).

The KEGG pathway map of the plant hormone biosynthesis consists of several sub-pathways. This figure shows the list and the expression patterns of transcripts in the *Conium maculatum* L. transcriptome assembly annotated to encode the enzymes of the Zeatin biosynthesis (KEGG ID path:map00908) of the plant hormone biosynthesis.

The expression values are shown in the square-root scale. The false discovery rate (FDR) q-values of the differential expression (between the organ indicated vs the rest of the organs) are shown with the "+" sign whenever Q-value < 0.1. Note that the differential expression statistics could not be calculated for most of the transcripts due to the lack of sample size (two replicates). The figure was created with *pheatmap* R package.

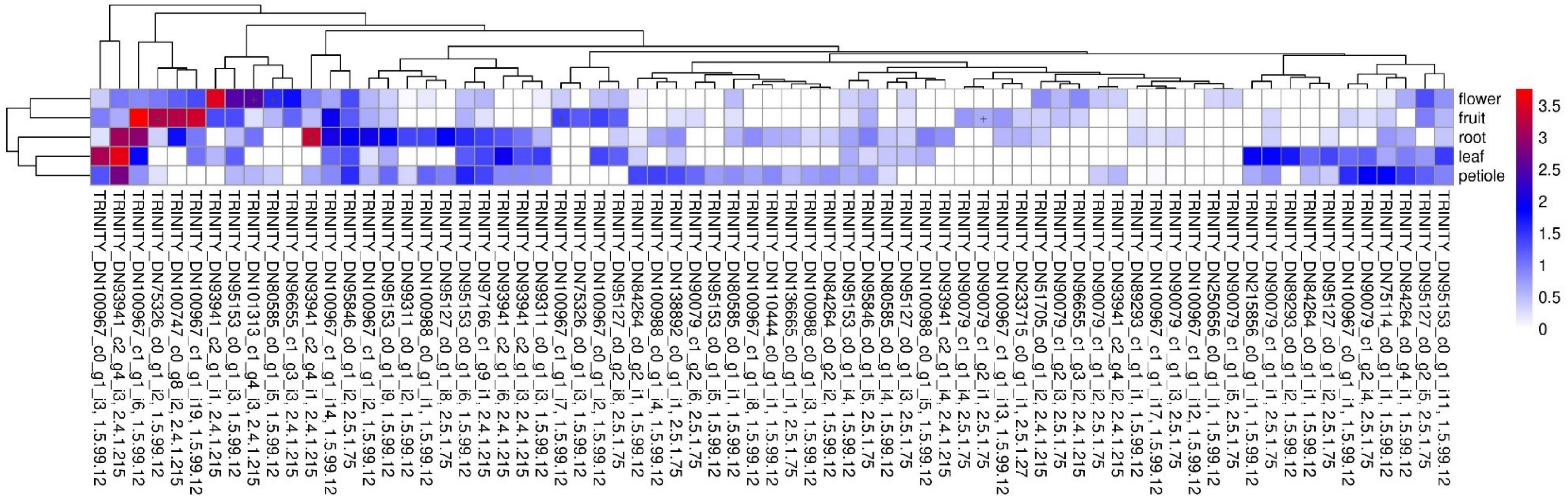

Supplementary Figure S7

Available online via https://doi.org/10.6084/m9.figshare.19783096 (NOTE: Because the image height exceeds the page height of this document, a rotated view of the figure is included below to minimize the loss of image quality. For better image quality, please check the online version).

The KEGG pathway map of the plant hormone biosynthesis consists of several sub-pathways. This figure shows the list and the expression patterns of transcripts in the *Conium maculatum* L. transcriptome assembly annotated to encode the enzymes of the biosynthesis of unsaturated fatty acids (KEGG ID path:map01040) of the plant hormone biosynthesis.

The expression values are shown in the square-root scale. The false discovery rate (FDR) q-values of the differential expression (between the organ indicated vs the rest of the organs) are shown with the "+" sign whenever Q-value < 0.1. Note that the differential expression statistics could not be calculated for most of the transcripts due to the lack of sample size (two replicates). The figure was created with *pheatmap* R package.

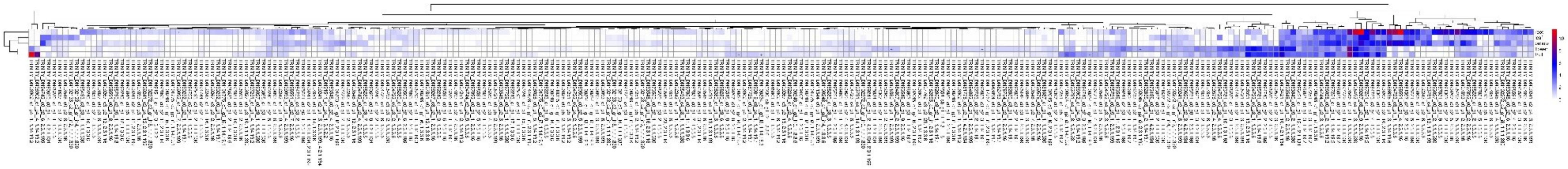

Supplementary Figure S8

Available online via https://doi.org/10.6084/m9.figshare.21154909

The expression profiling of selected gene candidates for *Conium* polyketide synthase 5 (CPKS5), polyketide reductase (PKR), L-alanine:5-keto-octanal aminotransferase (AAT), γ-coniceine reductase (CR), and *S*-adenosyl-L-methionine:coniine methyltransferase (CSAM) *in planta*. The relative expression of CPKS5, PKR1, AAT1, CR1, and CSAM1 with housekeeping genes glyceraldehyde-3-phosphate dehydrogenase (GADPH), cyclophilin 2 (CYP2) and actin (ACT). The expression values were calculated using $2^{-\Delta\Delta Ct}$-method and were normalized as follows. First, relative expression values were calculated with reference to leaf within each plant. Second, the relative expression values were normalized to CYP2 in subfigure **(a)** and GADPH in subfigure **(b)**. Error bars (ranges) were calculated based on two biological replicates with three technical replicates based on equation 1 (see Materials and Methods, Quantitative PCR section in the article's main text). The figure was drawn with *ggplot2* R package.

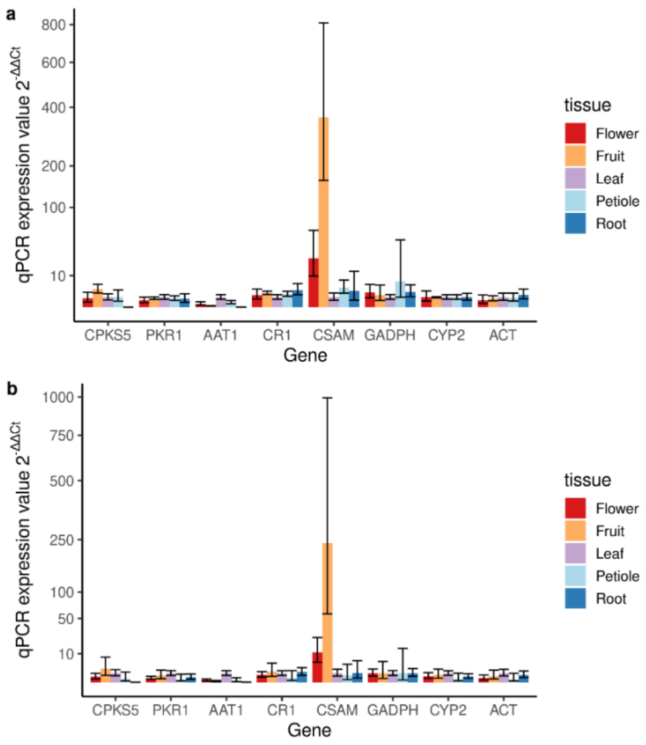

Supplementary Figure S9

Available online via https://doi.org/10.6084/m9.figshare.19761796

Co-expression clustering of the polyketide reductase (PKR) candidates found in sequence search (prior to the selection of the final set of candidates) along with CPKS5. The hierarchical clustering was computed based on the distances derived from correlation coefficients and the clustering was visualized as a heatmap. The figure was created with *pheatmap* R package.

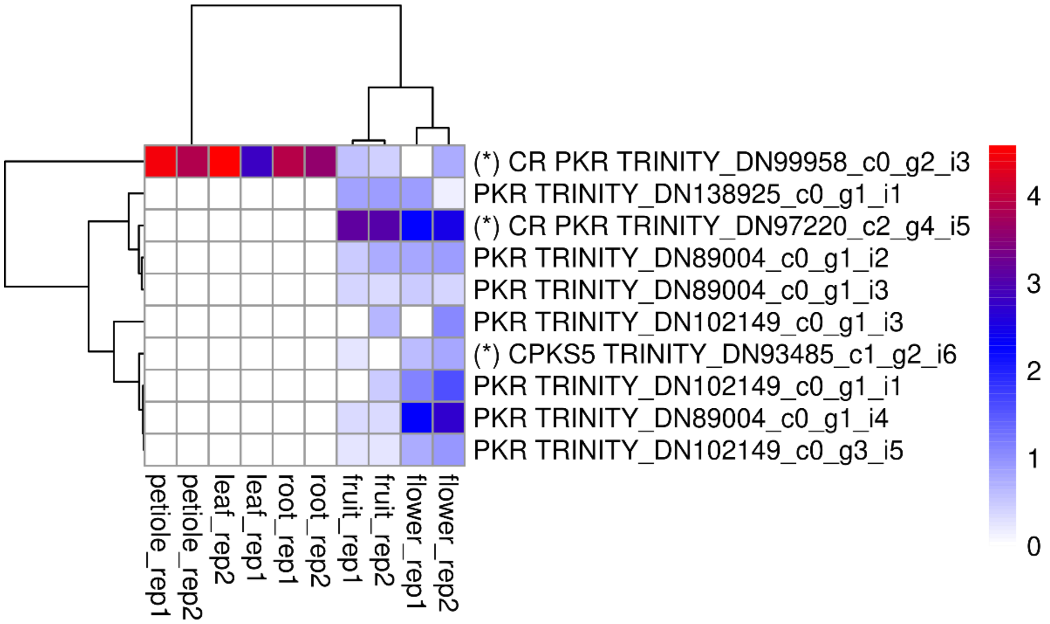

Supplementary Figure S10

Available online via https://doi.org/10.6084/m9.figshare.19778500

Co-expression clustering of the alanine aminotransferase (AAT) candidates found in sequence search (prior to the selection of the final set of candidates) along with CPKS5. The hierarchical clustering was computed based on the distances derived from correlation coefficients and the clustering was visualized as a heatmap. The figure was created with *pheatmap* R package.

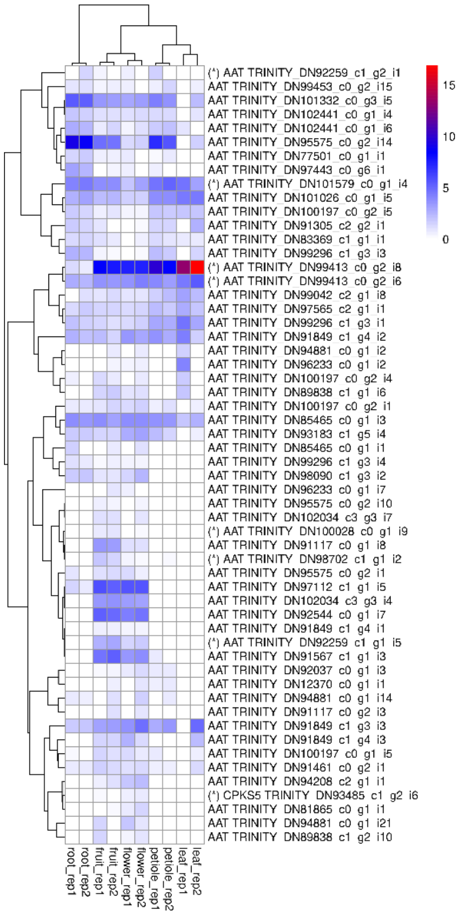

Supplementary Figure S11

Available online via https://doi.org/10.6084/m9.figshare.19778659 (NOTE: Because the image height exceeds the page height of this document, a rotated view of the figure is included below to minimize the loss of image quality).

Co-expression clustering of the γ-coniceine reductase (CR) candidates found in sequence search (prior to the selection of the final set of candidates) along with CPKS5. The hierarchical clustering was computed based on the distances derived from correlation coefficients and the clustering was visualized as a heatmap. The figure was created with *pheatmap* R package.

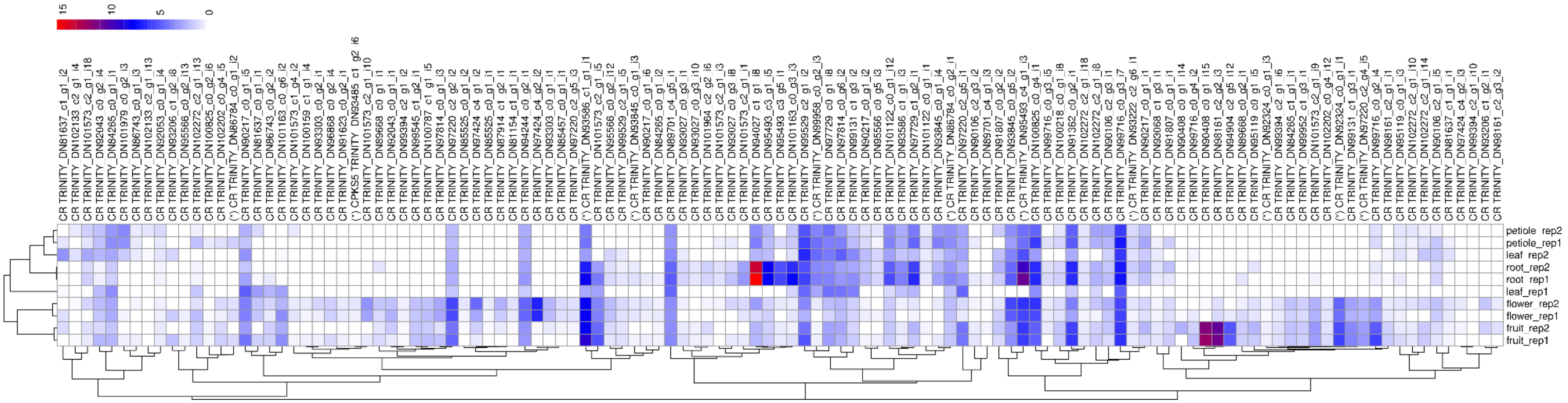

Supplementary Figure S12

Available online via https://doi.org/10.6084/m9.figshare.19778689

Co-expression clustering of the S-adenosyl-L-methionine:coniine methyltransferase (CSAM) candidates found in sequence search (prior to the selection of the final set of candidates) along with CPKS5. The hierarchical clustering was computed based on the distances derived from correlation coefficients and the clustering was visualized as a heatmap. The figure was created with *pheatmap* R package.

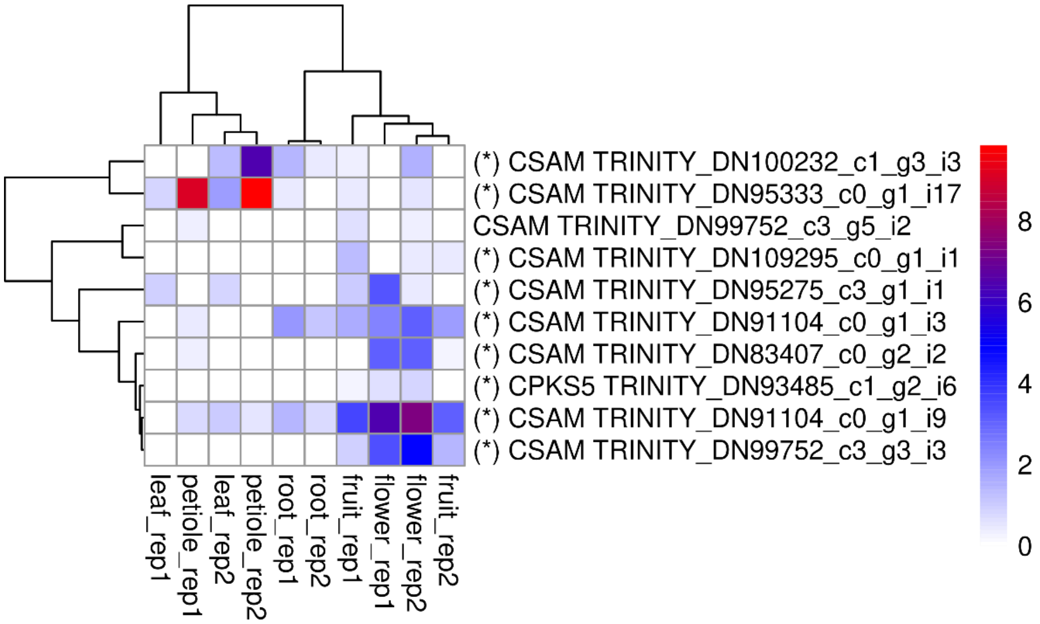

Supplementary Data S1

Available online as a FASTA file via https://doi.org/10.6084/m9.figshare.17308529

The FASTA file available online contains the sequences of all the 123 240 transcripts in the decontaminated transcriptome assembly and hence is not provided here inline.

Supplementary Data S2

Available online as an excel file with five sheets via https://doi.org/10.6084/m9.figshare.16900072

The Supplementary Data S3 available online is an excel file containing five sheets. Here, the five sheets will be listed as five different tables under sections named after the organ of the poison hemlock plant. These tables contain the full lists of Gene Ontology biological process terms that are found enriched in Conium maculatum organs, root, petiole, leaf, flower and developing fruit, using gene set enrichment analysis (GSEA; Subramanian, A. *et al.* Gene set enrichment analysis: a knowledge-based approach for interpreting genome-wide expression profiles. *Proc Natl Acad Sci U S A* **102**, 15545–15550 (2005)).

Flower

go_id	size	NES	pvalue	qvalue	go_name
GO:0009615	37	1.35057	0.003	1	response to virus
GO:1901072	43	1.33207	0.006	1	glucosamine-containing compound catabolic process
GO:0006026	43	1.32923	0.005	1	aminoglycan catabolic process
GO:0006032	43	1.32299	0.004	1	chitin catabolic process
GO:0046348	43	1.31387	0.008	1	amino sugar catabolic process
GO:0015977	46	1.36865	0.005	1	carbon fixation
GO:0016998	46	1.32581	0.008	1	cell wall macromolecule catabolic process
GO:0009607	52	1.31283	0.005	1	response to biotic stimulus
GO:0043094	63	1.32581	0.005	1	cellular metabolic compound salvage
GO:0009768	64	1.33726	0.001	1	photosynthesis, light harvesting in photosystem I
GO:0019253	71	1.29868	0.002	1	reductive pentose-phosphate cycle
GO:0009644	78	1.30599	0.004	1	response to high light intensity
GO:0000028	81	1.30886	0.003	1	ribosomal small subunit assembly
GO:0009617	83	1.26901	0.006	1	response to bacterium
GO:0071215	84	1.26427	0.004	1	cellular response to abscisic acid stimulus
GO:0009853	86	1.30757	0.004	1	photorespiration
GO:0000209	110	1.22588	0.007	0.95026	protein polyubiquitination
GO:0071396	112	1.24289	0.006	1	cellular response to lipid
GO:0050832	113	1.27188	0	1	defense response to fungus
GO:0000302	119	1.24235	0.006	0.98769	response to reactive oxygen species
GO:0006006	122	1.22318	0.008	0.92633	glucose metabolic process
GO:0009765	144	1.27911	0.003	1	photosynthesis, light harvesting
GO:0042742	161	1.23192	0.009	0.9706	defense response to bacterium
GO:0042273	179	1.20064	0.008	0.89604	ribosomal large subunit biogenesis
GO:0018298	185	1.27804	0	1	protein-chromophore linkage
GO:0043666	201	1.18409	0.009	0.87338	regulation of phosphoprotein phosphatase activity
GO:1901565	238	1.20321	0.003	0.90142	organonitrogen compound catabolic process

GO:0006091	248	1.19088	0.009	0.89174	generation of precursor metabolites and energy
GO:0002181	251	1.21779	0.002	0.91018	cytoplasmic translation
GO:0010876	256	1.32913	0	1	lipid localization
GO:0022900	261	1.18416	0.009	0.8757	electron transport chain
GO:0009409	263	1.21082	0.002	0.91123	response to cold
GO:0006869	272	1.31701	0	1	lipid transport
GO:0042221	276	1.24938	0	1	response to chemical
GO:0009737	288	1.1973	0.005	0.90325	response to abscisic acid
GO:0006950	294	1.2691	0	1	response to stress
GO:0070887	304	1.23968	0	0.98493	cellular response to chemical stimulus
GO:0006414	304	1.18805	0.007	0.89242	translational elongation
GO:0015979	307	1.29543	0	1	photosynthesis
GO:0000413	309	1.2231	0	0.92204	protein peptidyl-prolyl isomerization
GO:0018208	309	1.22096	0.001	0.91005	peptidyl-proline modification
GO:0018193	312	1.21879	0	0.91412	peptidyl-amino acid modification
GO:0009636	328	1.22921	0	0.95286	response to toxic substance
GO:0046686	377	1.17754	0.005	0.88385	response to cadmium ion
GO:0098754	379	1.21271	0	0.92714	detoxification
GO:0097237	406	1.20928	0	0.91871	cellular response to toxic substance
GO:1990748	411	1.20661	0	0.92009	cellular detoxification
GO:0098869	419	1.20564	0	0.90873	cellular oxidant detoxification
GO:0006979	452	1.19456	0	0.90465	response to oxidative stress
GO:0009651	461	1.17952	0.002	0.88036	response to salt stress

Fruit

go_id	size	NES	pvalue	qvalue	go_name
GO:0010344	16	1.44194	0.00401	1	seed oilbody biogenesis
GO:0048440	24	1.42706	0.003	1	carpel development

GO:0050826	29	1.41413	0.006	1	response to freezing
GO:0048509	26	1.40301	0.005	1	regulation of meristem development
GO:0010183	17	1.40044	0.00806	1	pollen tube guidance
GO:0019915	55	1.36214	0.009	1	lipid storage
GO:0010876	277	1.28485	0.008	1	lipid localization
GO:0006869	292	1.28403	0.008	1	lipid transport

Leaf

go_id	size	NES	pvalue	qvalue	go_name
GO:0042548	18	1.48904	0.00602	1	regulation of photosynthesis, light reaction
GO:0015977	38	1.47522	0.003	1	carbon fixation
GO:0009645	22	1.46315	0.004	1	response to low light intensity stimulus
GO:0043094	46	1.45565	0.001	1	cellular metabolic compound salvage
GO:0010114	41	1.44565	0.002	1	response to red light
GO:0010218	47	1.44315	0.005	1	response to far red light
GO:0009768	72	1.43277	0.004	1	photosynthesis, light harvesting in photosystem I
GO:0010207	39	1.43112	0.008	1	photosystem II assembly
GO:0010109	45	1.42485	0.002	1	regulation of photosynthesis
GO:0019253	66	1.41531	0.005	1	reductive pentose-phosphate cycle
GO:0009853	71	1.4075	0.003	1	photorespiration
GO:0018298	182	1.39558	0.003	1	protein-chromophore linkage
GO:0009765	159	1.39354	0.003	1	photosynthesis, light harvesting
GO:0015979	285	1.36041	0.009	1	photosynthesis
GO:0009416	149	1.33659	0.007	1	response to light stimulus

Root

go_id	size	NES	pvalue	qvalue	go_name
GO:0006542	16	1.42997	0.00402	1	glutamine biosynthetic process
GO:0050826	22	1.4316	0.00501	1	response to freezing
GO:1902652	35	1.36803	0.004	0.59903	secondary alcohol metabolic process
GO:0072350	37	1.37999	0.006	0.81126	tricarboxylic acid metabolic process
GO:0010466	40	1.38754	0.004	1	negative regulation of peptidase activity
GO:0045861	40	1.38676	0.005	1	negative regulation of proteolysis
GO:0052548	40	1.38427	0.006	0.97871	regulation of endopeptidase activity
GO:0010951	40	1.38111	0.006	0.84478	negative regulation of endopeptidase activity
GO:0006026	41	1.40105	0.001	1	aminoglycan catabolic process
GO:0046348	41	1.39532	0.002	1	amino sugar catabolic process
GO:0006032	41	1.3848	0.002	1	chitin catabolic process
GO:1901072	41	1.38216	0.008	0.88316	glucosamine-containing compound catabolic process
GO:0016998	43	1.38643	0.003	1	cell wall macromolecule catabolic process
GO:0006030	44	1.38325	0.003	0.924	chitin metabolic process
GO:0006102	44	1.32473	0.008	0.66167	isocitrate metabolic process
GO:1901071	46	1.39499	0.002	1	glucosamine-containing compound metabolic process
GO:0051346	46	1.35972	0.005	0.661	negative regulation of hydrolase activity
GO:0042044	46	1.33182	0.007	0.68718	fluid transport
GO:0052547	48	1.37786	0.002	0.79935	regulation of peptidase activity
GO:0006097	55	1.36324	0.004	0.63681	glyoxylate cycle
GO:0030162	62	1.3494	0.004	0.6337	regulation of proteolysis
GO:0006833	64	1.33008	0.004	0.67787	water transport
GO:0032515	66	1.28629	0.009	0.68163	negative regulation of phosphoprotein phosphatase activity
GO:0006094	66	1.28583	0.007	0.67247	gluconeogenesis
GO:0032269	69	1.37095	0.001	0.6846	negative regulation of cellular protein metabolic process
GO:0051248	69	1.37039	0.003	0.66456	negative regulation of protein metabolic process

GO:0071215	82	1.26611	0.008	0.67102	cellular response to abscisic acid stimulus
GO:0009415	83	1.30427	0.006	0.6929	response to water
GO:0044092	97	1.32836	0.001	0.67008	negative regulation of molecular function
GO:0043086	108	1.31987	0.004	0.66954	negative regulation of catalytic activity
GO:0006730	108	1.2675	0.009	0.6846	one-carbon metabolic process
GO:0016999	112	1.35198	0	0.63811	antibiotic metabolic process
GO:0071396	115	1.22762	0.007	0.68059	cellular response to lipid
GO:0042743	118	1.34573	0.001	0.64451	hydrogen peroxide metabolic process
GO:0042737	124	1.34529	0.003	0.63439	drug catabolic process
GO:0051187	131	1.34242	0	0.63682	cofactor catabolic process
GO:0017001	134	1.35077	0	0.63478	antibiotic catabolic process
GO:0042744	149	1.35551	0	0.63231	hydrogen peroxide catabolic process
GO:0097305	151	1.2373	0.009	0.68232	response to alcohol
GO:1901565	164	1.23299	0.007	0.68869	organonitrogen compound catabolic process
GO:0033993	164	1.22707	0.008	0.67848	response to lipid
GO:0072593	172	1.32632	0.001	0.65538	reactive oxygen species metabolic process
GO:0051172	176	1.25832	0.006	0.66149	negative regulation of nitrogen compound metabolic process
GO:0009179	178	1.21395	0.008	0.67071	purine ribonucleoside diphosphate metabolic process
GO:0002181	192	1.29031	0.001	0.66448	cytoplasmic translation
GO:0006099	197	1.24911	0.006	0.65777	tricarboxylic acid cycle
GO:0042221	217	1.2797	0.005	0.66535	response to chemical
GO:0006414	225	1.27779	0	0.65929	translational elongation
GO:0070887	244	1.26349	0.003	0.66902	cellular response to chemical stimulus
GO:0051336	253	1.18499	0.009	0.72527	regulation of hydrolase activity
GO:0001101	267	1.25524	0.003	0.66989	response to acid chemical
GO:0009636	270	1.2646	0.001	0.67448	response to toxic substance
GO:0006952	279	1.21863	0.007	0.68615	defense response
GO:0006096	298	1.20472	0.004	0.69366	glycolytic process
GO:0009737	312	1.18996	0.007	0.7205	response to abscisic acid

GO:0098754	314	1.26072	0	0.66398	detoxification
GO:0097237	340	1.25871	0.003	0.66242	cellular response to toxic substance
GO:1990748	344	1.25937	0	0.66602	cellular detoxification
GO:0098869	357	1.25147	0	0.66676	cellular oxidant detoxification
GO:0006979	413	1.29603	0	0.69994	response to oxidative stress
GO:0046686	422	1.18692	0.007	0.72684	response to cadmium ion
GO:0044265	453	1.20854	0.003	0.68256	cellular macromolecule catabolic process

Petiole

go_id	size	NES	pvalue	qvalue	go_name
GO:0045901	16	1.42558	0.00704	1	positive regulation of translational elongation
GO:0045905	16	1.42316	0.00902	1	positive regulation of translational termination
GO:0006452	16	1.42067	0.0091	1	translational frameshifting
GO:0042548	18	1.46848	0.00301	1	regulation of photosynthesis, light reaction
GO:0009645	19	1.43008	0.004	1	response to low light intensity stimulus
GO:0010167	24	1.41541	0.00901	0.83223	response to nitrate
GO:0034250	29	1.38961	0.009	0.83256	positive regulation of cellular amide metabolic process
GO:0006448	35	1.383	0.005	0.76776	regulation of translational elongation
GO:1901071	40	1.42573	0.003	1	glucosamine-containing compound metabolic process
GO:0006032	40	1.42485	0.001	1	chitin catabolic process
GO:1901072	40	1.41906	0.003	1	glucosamine-containing compound catabolic process
GO:0006030	40	1.41633	0.006	0.95409	chitin metabolic process
GO:0006026	40	1.41179	0.003	0.83481	aminoglycan catabolic process
GO:0046348	40	1.40927	0.005	0.8251	amino sugar catabolic process
GO:0046500	40	1.37034	0.00501	0.71748	S-adenosylmethionine metabolic process
GO:0042542	40	1.36946	0.008	0.7093	response to hydrogen peroxide

GO:0016998	41	1.42505	0.001	1	cell wall macromolecule catabolic process
GO:0009615	41	1.41544	0.002	0.89549	response to virus
GO:0045727	41	1.33284	0.009	0.69678	positive regulation of translation
GO:0042044	43	1.40329	0.006	0.74422	fluid transport
GO:0010207	44	1.37782	0.005	0.78307	photosystem II assembly
GO:0043094	46	1.39654	0.002	0.76551	cellular metabolic compound salvage
GO:0015977	54	1.40924	0.003	0.77686	carbon fixation
GO:0042255	55	1.32359	0.008	0.72764	ribosome assembly
GO:0006833	59	1.37755	0.003	0.73718	water transport
GO:0009768	66	1.41898	0.004	0.98276	photosynthesis, light harvesting in photosystem I
GO:0016999	77	1.37766	0.003	0.75919	antibiotic metabolic process
GO:0009853	77	1.37455	0.004	0.75267	photorespiration
GO:0009266	79	1.33101	0.004	0.69364	response to temperature stimulus
GO:0042743	81	1.37255	0.006	0.73226	hydrogen peroxide metabolic process
GO:0009644	89	1.32778	0.009	0.69728	response to high light intensity
GO:0017001	92	1.38099	0.003	0.76767	antibiotic catabolic process
GO:0042737	96	1.35683	0.008	0.76395	drug catabolic process
GO:0051187	104	1.36713	0.004	0.68245	cofactor catabolic process
GO:0006730	109	1.30911	0.004	0.73085	one-carbon metabolic process
GO:0042744	118	1.38378	0.002	0.84782	hydrogen peroxide catabolic process
GO:0072593	130	1.35588	0.008	0.7416	reactive oxygen species metabolic process
GO:0009735	134	1.27309	0.007	0.73784	response to cytokinin
GO:0009765	152	1.40441	0	0.7675	photosynthesis, light harvesting
GO:0009408	165	1.30539	0.009	0.74056	response to heat
GO:0006414	166	1.3031	0.006	0.72983	translational elongation
GO:0002181	170	1.29567	0.005	0.71749	cytoplasmic translation
GO:0042221	178	1.32161	0.007	0.71768	response to chemical
GO:0018298	191	1.40154	0.001	0.73175	protein-chromophore linkage
GO:0070887	197	1.30991	0.003	0.74011	cellular response to chemical stimulus
GO:0009636	217	1.31775	0.006	0.70021	response to toxic substance

GO:0098754	259	1.30847	0.009	0.7287	detoxification
GO:0098869	289	1.29726	0.008	0.73113	cellular oxidant detoxification
GO:0015979	296	1.34758	0.007	0.75537	photosynthesis
GO:0006979	335	1.30914	0.002	0.73901	response to oxidative stress
GO:0046686	391	1.21485	0.005	0.72381	response to cadmium ion

Supplementary Data S3

Available online as Word document via https://doi.org/10.6084/m9.figshare.17061713

The amino acid sequences encoded by the two CPKS5 transcript candidates found in this study are aligned with the CPKS5 sequence reported in Hannu et al., 2015 study (Hotti, H., Seppänen-Laakso, T., Arvas, M., Teeri, T. H. & Rischer, H. Polyketide synthases from poison hemlock (*Conium maculatum* L.). *FEBS J.* **282**, 4141–4156 (2015)). The sequence alignment is shown below as plain text.

```
                                                  20                  40
                              ....|....|....|....|....|....|....|....|....|....|
TRINITY_DN93485_c1_g2_i6      MVTVNEFRKAQQAEGPATVLAIGTATPPNCVDQSTYADYYFRVTKSEDKT
TRINITY_DN93485_c1_g2_i1      MVTVNEFRKAQQAEGPATVLAIGTATPPNCVDQSTYADYYFRVTKSEDKT
CPKS5                         MVTVNEFRKAHLAEGPATVLAIGTATPSYCIDQSTFPDLYFRTTKSEDET
                              **********: ***************. *:****:.* ***.*****:*

                                      60                  80                 100
                              ....|....|....|....|....|....|....|....|....|....|
TRINITY_DN93485_c1_g2_i6      ELKEKFKRMCDRSMINTRYTHLTEEFMKENPDFWNMAPSLDARQEIVVNE
TRINITY_DN93485_c1_g2_i1      ELKEKFKRMCDRSMINTRYTHLTEEFMKENPDFWNMAPSLDARQEIVVNE
CPKS5                         ELKEKFKRMCDRSMINTRYTHLTEEFMKENPDFWNMAPSLDARQEIVVNE
                              **************************************************

                                                 120                 140
                              ....|....|....|....|....|....|....|....|....|....|
TRINITY_DN93485_c1_g2_i6      VPKLGKEAATKAIKEWGQPMSKITHLVFCTYSSADFPGADFRLTKLLGLS
TRINITY_DN93485_c1_g2_i1      VPKLGKEAATKAIKEWGQPMSKITHLVFCTYSSADFPGADFRLTKLLGLS
CPKS5                         VPKLGKEAATKAIKEWGQPMSKITHLVFCTYSSADFPGADFRLTKLLGLS
                              **************************************************

                                     160  ▲              180                 200
                              ....|....|....|....|....|....|....|....|....|....|
TRINITY_DN93485_c1_g2_i6      PSVKRSMLYQQGCFAGGTGLRLAKDLAENNKGARVLVVCSELSVLAFQGP
TRINITY_DN93485_c1_g2_i1      PSVKRSMLYQQGCFAGGTGLRLAKDLAENNKGARVLVVCSELSVLAFQGP
CPKS5                         PSVKRSMLYQQGCFAGGTGLRLAKDLAENNKGARVLVVCSELSVLAFQGP
                              **************************************************

                                                 220                 240
                              ....|....|....|....|....|....|....|....|....|....|
TRINITY_DN93485_c1_g2_i6      KVIDIDCLITQALFGDGAVAVIVGSDPVIGVEKPLFEIFSAAQTIIPDSD
TRINITY_DN93485_c1_g2_i1      KVIDIDCLITQALFGDGAVAVIVGSDPVIGVEKPLFEIFSAAQTIIPDSD
```

```
CPKS5                         KVIDIDCLITQALFGDGAVAVIVGSDPVIGVEKPLFEIFSAAQTIIPDSD
                              **************************************************

                                     260                 280                 300
                              ....|....|....|....|....|....|....|....|....|....|
TRINITY_DN93485_c1_g2_i6      GAIKGYLRKVGLTFHLRKDVPGLIAKNIRKYLVEAFQPLGITDWNSIFWI
TRINITY_DN93485_c1_g2_i1      GAIKGYLRKVGLTFHLRKDVPGLIAKNIRKYLVEAFQPLGITDWNSIFWI
CPKS5                         GAIKGYLRKVGLTFHLRKDVPGLIAKNIRKYLVEAFQPLGITDWNSIFWI
                              **************************************************

                               ▲               320              ▲  340
                              ....|....|....|....|....|....|....|....|....|....|
TRINITY_DN93485_c1_g2_i6      AHPGGPAILDQIEKELSLKPEKLKSTRQVLRDYGNLSSASVLFILDEMRK
TRINITY_DN93485_c1_g2_i1      AHPGGPAILDQIEKELSLKPEKLKSSRQILRDYGNLSSASVLFILDEMRK
CPKS5                         AHPGGPAILDQIEKELSLKPEKLKSSRQILRDYGNLSSASVLFIMDEMRK
                              *************************:**:***************:*****

                                     360                 380
                              ....|....|....|....|....|....|....|....|....|....|
TRINITY_DN93485_c1_g2_i6      ASAKDGKRSTGEGLDWGVLFGFGPGLTVETVVLHSVPT
TRINITY_DN93485_c1_g2_i1      ASAKDGKPTTGEGLDWGVLFGFGPGLTVETVVLHSVPT
CPKS5                         ASAKDGKPTTGEGLDWGVLFGFGPGLTVETVVLHSVPT
                              ******* :*****************************
```

Supplementary Data S4

Available online as two FASTA files via https://doi.org/10.6084/m9.figshare.12738578

The Supplementary Data S4 (the data set containing aminotransferase sequences used for identifying the aminotransferase candidates in the poison hemlock transcriptome) is provided online-only at the web address indicated above. This data set contains two FASTA files: (1) uniprot_transaminases.fasta: the set of transaminase sequences retrieved from the Uniprot database, and (2) pdb_transaminases.fasta: the set of transaminase sequences retrieved from the protein data bank (PDB) database. In order to find the alanine aminotransferase enzyme involved in the coniine biosynthesis in the poison hemlock transcriptome, these sequences were used as the seeds for homology-based search.

References

1. Wen, J. *et al.* A transcriptome-based study on the phylogeny and evolution of the taxonomically controversial subfamily Apioideae (Apiaceae). *Ann Bot* **125**, 937–953 (2020).
2. Li, M. Y., Wang, F., Jiang, Q., Ma, J. & Xiong, A. S. Identification of SSRs and differentially expressed genes in two cultivars of celery (Apium graveolens L.) by deep transcriptome sequencing. *Hortic Res* **1**, 1–9 (2014).
3. Sangwan, R. S., Tripathi, S., Singh, J., Narnoliya, L. K. & Sangwan, N. S. De novo sequencing and assembly of Centella asiatica leaf transcriptome for mapping of structural, functional and regulatory genes with special reference to secondary metabolism. *Gene* **525**, 58–76 (2013).
4. Galata, M., Sarker, L. S. & Mahmoud, S. S. Transcriptome profiling, and cloning and characterization of the main monoterpene synthases of Coriandrum sativum L. *Phytochemistry* **102**, 64–73 (2014).
5. Tan, G. F. *et al.* De novo assembly and transcriptome characterization: novel insights into the temperature stress in Cryptotaenia japonica Hassk. *Acta Physiol Plant* **37**, 1–12 (2015).
6. Xu, Z.-S., Tan, H.-W., Wang, F., Hou, X.-L. & Xiong, A.-S. CarrotDB: a genomic and transcriptomic database for carrot. *Database* **2014**, bau096–bau096 (2014).
7. Amini, H. *et al.* Tissue-Specific Transcriptome Analysis Reveals Candidate Genes for Terpenoid and Phenylpropanoid Metabolism in the Medicinal Plant Ferula assafoetida. *G3 Genes|Genomes|Genetics* **9**, 807–816 (2019).

8. Sobhani Najafabadi, A., Naghavi, M. R., Farahmand, H. & Abbasi, A. Transcriptome and metabolome analysis of Ferula gummosa Boiss. to reveal major biosynthetic pathways of galbanum compounds. *Funct Integr Genomics* **17**, 725–737 (2017).

9. Song, T. *et al.* Comparative transcriptome of rhizome and leaf in Ligusticum Chuanxiong. *Plant Syst Evol 2015 3018* **301**, 2073–2085 (2015).

10. Jia, Y. *et al.* Comparative Transcriptome Analysis Reveals Adaptive Evolution of Notopterygium incisum and Notopterygium franchetii, Two High-Alpine Herbal Species Endemic to China. *Molecules* **22**, 1158 (2017).

11. Li, M.-Y. *et al.* De Novo Transcriptome Sequence Assembly and Identification of AP2/ERF Transcription Factor Related to Abiotic Stress in Parsley (Petroselinum crispum). *PLoS One* **9**, e108977 (2014).

12. Drew, D. *et al.* Transcriptome Analysis of Thapsia laciniata Rouy Provides Insights into Terpenoid Biosynthesis and Diversity in Apiaceae. *Int J Mol Sci* **14**, 9080–9098 (2013).

13. Amiripour, M., Sadat Noori, S. A., Shariati, V. & Soltani Howyzeh, M. Transcriptome analysis of Ajowan (Trachyspermum ammi L.) inflorescence. *J Plant Biochem Biotechnol 2019 284* **28**, 496–508 (2019).

14. Kanehisa, M. Toward understanding the origin and evolution of cellular organisms. *Protein Sci* **28**, 1947–1951 (2019).

15. Darzi, Y., Letunic, I., Bork, P. & Yamada, T. iPath3.0: interactive pathways explorer v3. *Nucleic Acids Res* **46**, W510–W513 (2018).
